# Mechanism of efficient double-strand break repair by a long non-coding RNA

**DOI:** 10.1093/nar/gkaa784

**Published:** 2020-10-12

**Authors:** Roopa Thapar, Jing L Wang, Michal Hammel, Ruiqiong Ye, Ke Liang, Chengcao Sun, Ales Hnizda, Shikang Liang, Su S Maw, Linda Lee, Heather Villarreal, Isaac Forrester, Shujuan Fang, Miaw-Sheue Tsai, Tom L Blundell, Anthony J Davis, Chunru Lin, Susan P Lees-Miller, Terence R Strick, John A Tainer

**Affiliations:** Department of Molecular and Cellular Oncology, University of Texas M.D. Anderson Cancer Center, Houston, TX 77030, USA; Ecole Normale Supérieure, IBENS, CNRS, INSERM, PSL Research University, Paris 75005, France; Molecular Biophysics and Integrated Bioimaging, Lawrence Berkeley National Laboratory, 1 Cyclotron Rd, Berkeley, CA 94720, USA; Department of Biochemistry and Molecular Biology, Robson DNA Science Centre, Charbonneau Cancer Institute, University of Calgary, Alberta, T2N 4N1, Canada; Department of Molecular and Cellular Oncology, University of Texas M.D. Anderson Cancer Center, Houston, TX 77030, USA; Department of Molecular and Cellular Oncology, University of Texas M.D. Anderson Cancer Center, Houston, TX 77030, USA; Department of Biochemistry, University of Cambridge, 80 Tennis Court Road, Cambridge CB2 1GA, UK; Department of Biochemistry, University of Cambridge, 80 Tennis Court Road, Cambridge CB2 1GA, UK; Biological Systems and Bioengineering, Lawrence Berkeley National Laboratory, Berkeley, CA, 94720, USA; Department of Biochemistry and Molecular Biology, Robson DNA Science Centre, Charbonneau Cancer Institute, University of Calgary, Alberta, T2N 4N1, Canada; CryoEM Core at Baylor College of Medicine, Houston, Texas 77030, USA; CryoEM Core at Baylor College of Medicine, Houston, Texas 77030, USA; Department of Biochemistry and Molecular Biology, Robson DNA Science Centre, Charbonneau Cancer Institute, University of Calgary, Alberta, T2N 4N1, Canada; Biological Systems and Bioengineering, Lawrence Berkeley National Laboratory, Berkeley, CA, 94720, USA; Department of Biochemistry, University of Cambridge, 80 Tennis Court Road, Cambridge CB2 1GA, UK; Division of Molecular Radiation Biology, Department of Radiation Oncology, UT Southwestern Medical Center, Dallas, TX 75390, USA; Department of Molecular and Cellular Oncology, University of Texas M.D. Anderson Cancer Center, Houston, TX 77030, USA; Department of Biochemistry and Molecular Biology, Robson DNA Science Centre, Charbonneau Cancer Institute, University of Calgary, Alberta, T2N 4N1, Canada; Ecole Normale Supérieure, IBENS, CNRS, INSERM, PSL Research University, Paris 75005, France; Programme “Equipe Labellisée’’, Ligue Nationale Contre le Cancer, Paris 75005, France; Department of Molecular and Cellular Oncology, University of Texas M.D. Anderson Cancer Center, Houston, TX 77030, USA; Molecular Biophysics and Integrated Bioimaging, Lawrence Berkeley National Laboratory, 1 Cyclotron Rd, Berkeley, CA 94720, USA; Department of Cancer Biology, University of Texas M.D. Anderson Cancer Center, Houston, TX 77030, USA

## Abstract

Mechanistic studies in DNA repair have focused on roles of multi-protein DNA complexes, so how long non-coding RNAs (lncRNAs) regulate DNA repair is less well understood. Yet, lncRNA LINP1 is over-expressed in multiple cancers and confers resistance to ionizing radiation and chemotherapeutic drugs. Here, we unveil structural and mechanistic insights into LINP1’s ability to facilitate non-homologous end joining (NHEJ). We characterized LINP1 structure and flexibility and analyzed interactions with the NHEJ factor Ku70/Ku80 (Ku) and Ku complexes that direct NHEJ. LINP1 self-assembles into phase-separated condensates via RNA–RNA interactions that reorganize to form filamentous Ku-containing aggregates. Structured motifs in LINP1 bind Ku, promoting Ku multimerization and stabilization of the initial synaptic event for NHEJ. Significantly, LINP1 acts as an effective proxy for PAXX. Collective results reveal how lncRNA effectively replaces a DNA repair protein for efficient NHEJ with implications for development of resistance to cancer therapy.

## INTRODUCTION

Non-homologous end-joining (NHEJ) is the major pathway for repair of ionizing radiation induced double-strand breaks (DSBs) during G0 and in the G1 phase of the cell cycle in mammalian cells (reviewed in (1–5). NHEJ involves an initial synaptic event in which the two DNA ends are brought together by the human Ku protein. Ku is a heterodimer of 70 and 83 kDa polypeptides (Ku70/80) that serves as the DNA-binding subunit of the DNA-dependent protein kinase (DNA-PK), binding tightly to ends of double stranded (ds) DNA *in vitro* (6–10). Besides detecting and binding DSB ends and activating DNA-PKcs, Ku is required for recruitment of other NHEJ factors to the break, including XRCC4 (11), XLF (12), APLF (13), PAXX (14) and the cell cycle regulator CYREN/MRI (15) and is indispensable for NHEJ. Ku can also bind RNA (16–18) and recent studies suggest that DNA-PK activity (which comprises of Ku and DNA-PK catalytic subunit, DNA-PKcs) may be required for diverse processes in RNA metabolism that include regulation of rRNA processing and haematopoiesis (19), the cGAS-STING innate immune pathway (20) and atherosclerosis (21).

LncRNAs are >200 nt RNA Pol-II transcripts that generally are not translated into proteins (22–27). Notably, it is projected that >60 000 lncRNAs are expressed in humans, i.e. greater than the number of protein-coding genes in the human genome. Several lncRNAs have already been implicated in disease states (28,29). The long non-coding RNA ‘lncRNA in non-homologous end joining pathway’ (LINP1) (Supplementary Figure S1A) was identified as a lncRNA that enhances DNA repair via the NHEJ pathway in triple negative breast cancers (TNBCs) (30). Knockdown of LINP1 sensitized mice to ionizing radiation due to defects in the NHEJ DNA repair pathway. LINP1 is a 917 nt ncRNA transcribed from two exons that are highly conserved in primates (Supplementary Figures S1A, S1B and S2). Biochemical and cell biological data (30) suggested that LINP1 may act as a novel RNA factor to facilitate the interaction between Ku70/80 and DNA-PKcs during DNA repair via NHEJ in TNBC tumors, but the mechanism by which it acts remained to be defined. Since the original study (30), the role of LINP1 in breast cancer metastasis and its association with Ku and DNA-PKcs has been supported by other reports (31–35). LINP1 is a p53-linked lncRNA and high expression of LINP1 is correlated with poor prognosis and patient survival outcomes. LINP1 levels are also upregulated in cervical cancer (32), prostate cancer (36) and lung cancer (37), suggesting that the LINP1 RNA-dependent repair mechanism, which facilitates NHEJ in tumors, is utilized broadly in multiple cancer sub-types.

We show here that full-length LINP1 is flexible and does not form a compact structure in solution. The RNA itself can form phase-separated condensates that reorganize into long filamentous structures upon Ku association. Ku binds several stem-loops and a G-quadruplex from LINP1 RNA with comparable affinities. LINP1 does not affect DNA-PK’s ability to phosphorylate XLF, although our small-angle X-ray scattering (SAXS) data indicate that the RNA- and DNA-binding sites overlap on Ku. Size exclusion chromatography SAXS (SEC-SAXS) results suggest that interaction with LINP1 RNA increases association between two Ku heterodimers. Surprisingly, by single-molecule nanomanipulation (38), we find that LINP1 is more effective than PAXX for increasing the lifetime of the initial synaptic event during NHEJ. Furthermore, we show that Ku interacts with LINP1 RNA in the presence of XLF and APLF but not PAXX, suggesting that in cells LINP1 may act to fulfill the role of PAXX, pre-organizing a scaffold with NHEJ components for regulating end synapsis. Taken together, our results reveal that LINP1 lncRNA is capable of recruiting multiple Ku-NHEJ assemblies via an extended scaffold and structured motifs. LINP1 substitutes for PAXX efficiently by (i) increasing the net concentration of NHEJ factors at the double strand break via Ku and (ii) bridging two Ku heterodimers across the double strand break to promote DNA end-joining in NHEJ in cancer cells. Importantly, these data establish roles for lncRNAs in effectively replacing proteins in the DNA damage response with implications for cancer etiology, prognosis and therapeutic strategies.

## MATERIALS AND METHODS

### Purification of proteins

DNA-PKcs and Ku70/80 were purified from HeLa cells as described (39,40). Full-length Ku70/His-Ku80 heterodimer (Ku70/Ku80 1–718), Ku ΔCTR (Ku70/Ku80 1–569), Ku 5D and Ku 5A mutants were expressed and purified from baculovirus-infected insect cells as described (41). Full-length PAXX was purified as a GST fusion protein, cleaved from the GST tag using PreScission protease and further purified on a Heparin column for SAXS studies using standard protocols.

### Preparation of RNA

A plasmid containing the LINP1 gene was custom synthesized in a pUC vector (Genscript) with a T7 promoter. Full-length LINP1, nts 1–300, 301–600 and 601–917 RNA fragments were amplified using PCR, the PCR fragments were purified using a PCR cleanup kit (Qiagen) and used as templates for *in vitro* transcription for 2 h at 37°C using standard protocols. Following transcription, the reaction was treated with RNase-free DNase for 30 min at 37°C to remove the template DNA followed by treatment with proteinase K at 37°C for 1 h. The RNA samples were folded by the addition of 3.3× folding buffer (333 mM Hepes pH 8.0, 20 mM MgCl_2_, 333 mM NaCl), snap cooled on ice for 10 min after heating to 95°C for 5 min, followed by a slow refolding at 37°C for 60 min. We tried different protocols with and without snap cooling as well as varied the concentration of MgCl_2_ to optimize folding conditions for the RNA. RNA samples were concentrated and loaded onto a size exclusion column (either Superose 6 or Superdex S200, depending on RNA size) in 100 mM Hepes pH 8.0, 5 mM MgCl_2_, 100 mM NaCl and 20 mM KCl, to separate misfolded and truncated RNA species. RNA samples were never frozen, but were kept either at room temperature or at 4°C in the presence of RNase inhibitors.

Short unlabeled and 5′FAM labeled RNAs were chemically synthesized and deprotected by Dharmacon Research, Boulder, CO, USA or were synthesized by Integrated DNA Technologies (IDT). All RNAs were synthesized with a 5′-FAM group. The lower strand of the 25bp duplex DNA was synthesized with a 3′-FAM and annealed as described. The RNA samples were taken up in 20 mM potassium phosphate buffer, 20 mM KCl and 0.02 mM EDTA (pH 6.8). The RNA hairpins were folded by being heating at 95°C for 2 min and then snap-cooled on ice for 10 min to ensure the lowest-energy hairpin conformer for all studies. The formation of the stem-loop structure (instead of a duplex), using this approach was confirmed by NMR and circular dichroism. The effect of Mg^2+^ on the folding of the SL2 and SL3 RNAs was monitored using SAXS and SEC-MALS. The LINP1 and TERRA G-quadruplexes were annealed by heating the samples at 90°C for 5 min, followed by slow annealing at room temperature overnight.

### Electrophoretic mobility shift assays (EMSAs)

FAM-labeled RNA oligonucleotides were resuspended in 1× EMSA DNA-binding buffer (see below) to 100 μM and stored at −20°C until use. Purified proteins were incubated with FAM-labeled dsDNA or RNA in 1× DNA-binding buffer (25 mM Hepes-KOH, pH 7.5, 50 mM NaCl, 1 mM DTT, 1 mM EDTA and 10% glycerol) plus 0.1 μM BSA in a final volume of 20 μl. Samples were incubated at room temperature for 25 min in the dark and analyzed on 8% acrylamide non-denaturing gels in EMSA running buffer (50 mM Tris, 380 mM glycine, 2 mM EDTA) at 100 V for 55 min in the dark at RT. Gels were imaged using a Fuji Luminescent Image reader model # LAS-4000 and images were imported into Photoshop as tif files. Images were quantitated using ImageQuant 5.2 software (Molecular Dynamics).

### Size exclusion chromatography and multi-angle light scattering (SEC-MALS)

RNA samples (at 4 mg/ml) were loaded onto a GE Superdex 200 size exclusion column (24 ml) that was pre-equilibrated in 20 mM potassium phosphate buffer (pH 7.0) and 70 mM KCl. The eluted samples passed through a Wyatt DynaPro NanoStar dynamic light scattering (DLS) system, a Wyatt MALS (DAWN-Helios-II) system, and an Optilab T-rEX Refractive Index detector. The data were analyzed using ASTRA 7.0 software (Wyatt Technology). BSA (2 mg/ml) was used for normalization, delay time determination and band broadening correction in the ASTRA software.

### Circular dichroism (CD)

All CD experiments were conducted at 25°C using a JASCO J-810 CD spectrometer equipped with a Peltier temperature controller. The concentrations of the RNAs were 5 μM for CD spectra and 50 μM for the UV thermal denaturation experiments, in 20 mM potassium phosphate buffer, 20 mM KCl and 0.02 mM EDTA (pH 6.8). The CD spectra were recorded from 190–40 nm. For the UV melting experiments, the samples were heated from 20 to 90°C at a rate of 1°C min^−1^ and the UV absorbance at 263 nm was monitored. Data analysis to obtain the *T*_m_ was performed using Prism 4.3 (GraphPad).

### NMR spectroscopy

RNA samples were taken up in 20 mM potassium phosphate buffer, 20 mM KCl and 0.02 mM EDTA (pH 6.8). One-dimensional (1D) ^1^H NMR experiments for detection of RNA imino protons were conducted either on a Varian Inova 600 MHz spectrometer or on a Varian 600 MHz spectrometer with a 5 mm cryogenic ^1^H{^13^C/^15^N} probe. Each experiment was conducted with a binomial 1−1 echo pulse with maximal excitation at 12.5 ppm for solvent suppression. To confirm that the hairpins were folded, 2D ^1^H−^1^H NOESY spectra were recorded on 0.5 mM RNA samples with a mixing time of 125 ms at 10°C.

### Microscale thermophoresis (MST)

Microscale thermophoresis (MST) experiments were conducted in triplicate on a Monolith NT.115 system (NanoTemper Technologies) using standard treated capillary tubes. All data were collected at 24°C. 5′FAM-labeled RNAs were used at a concentration of 40 nM for all experiments. A 1:1 dilution series of the full-length Ku70/Ku80 heterodimer ligand was prepared in 50 mM Tris pH 8.0, 50 mM NaCl, 1 mM DTT, with the final concentrations of the ligands ranging from 93 μM to 2.8 nM. Samples were incubated on ice in the dark for 30 min. Following incubation, 10 μl samples were added to standard coated capillaries (NanoTemper Technologies) and were subjected to MST analysis. Thermophoresis of the labeled RNA was calculated for all samples, the results were analyzed, and the values obtained in triplicate were averaged, normalized and plotted against the ligand concentration. The dissociation constant *K*_D_ was determined using a single site model to fit the curve.

### High-throughput small angle X-ray scattering (HT-SAXS)

SAXS data on RNA samples were collected at the ALS (Berkeley) SIBYLS Beamline 12.3.1 using exposure times of 0.5, 1, 2 and 4 s on three sample concentrations (1, 2 and 5 mg/ml). Intensities from the buffer data were subtracted from the sample data. Scattering curves were merged using PRIMUS (42). Guinier plot analysis and radius of gyration (*R*_g_) were evaluated using PRIMUS and using the entire scattering curve with GNOM (43) separately. The pair-distance distribution function *P*(*r*) and maximal particle size (*D*_max_) were analyzed using GNOM. To determine *D*_max_, *P*(*r*) was computed while constraining the function to go to zero at *r*_max_, where *r*_max_ was varied from 100 to 200 Å. The Kratky plot, Porod-debye plot and Porod-debye value were calculated using the program SCÅTTER.

### SAXS and MALS data acquisition in line with size-exclusion chromatography (SEC-SAXS-MALS)

For small angle X-ray scattering coupled with multi-angle light scattering in line with size-exclusion chromatography (SEC-SAXS-MALS) experiments, 60 μl samples containing either 5 mg/ml (36 μM) of Ku delta CTR, 5 mg/ml (113 μM) PAXX dimer, and a mixture of 36 μM Ku delta CTR and 36 μM LINP1 SL2 RNA and 36 μM Ku delta CTR and 3.6 μM LINP1 FL RNA were prepared in 50 mM Hepes 7.5, 50 mM KCl, 5 mM MgCl_2_, 5% glycerol and 0.2 mM DTT. SEC-SAXS-MALS were collected at the ALS beamline 12.3.1 LBNL Berkeley, California (44). X-ray wavelength was set at *λ* = 1.127 Å and the sample-to-detector distance was 2100 mm resulting in scattering vectors, *q*, ranging from 0.01 to 0.4 Å^-1^. The scattering vector is defined as *q* = 4πsinθ/*λ*, where 2θ is the scattering angle. All experiments were performed at 20°C (45) and data were processed as described (46). Briefly, a SAXS flow cell was directly coupled with an online Agilent 1260 Infinity HPLC system using a Shodex KW803 column. The column was equilibrated with running buffer with a flow rate of 0.5 ml/min. About 55 μl of each sample was run through the SEC and 3-s X-ray exposures were collected continuously during a 30 min elution. The SAXS frames recorded prior to the protein elution peak were used to subtract all other frames. The subtracted frames were investigated by the radius of gyration *R*_g_ derived by the Guinier approximation *I*(*q*) = *I*(0) exp(-*qR*_g_)^2^/3 with the limits *qR*_g_ < 1.5. The elution peak was mapped by comparing integral of ratios to background and *R*_g_ relative to the recorded frame using the program SCÅTTER. Final merged SAXS profiles where used for further analysis including Guinier plot, which determined an aggregation free state (Supplementary Figures S12 and S13). The program SCÅTTER was used to compute the pair distribution function *P*(*r*). The distance *r* where *P*(*r*) approach zero intensity identifies the maximal dimension of the macromolecule (*D*_max_). *P*(*r*) functions were normalized based on the molecular weight of the assemblies as determined by SCÅTTER using volume of correlation *V*_c_ (47,48). The eluent was subsequently split 4 to 1 between SAXS line and a series of UV at 280 and 260 nm, multi-angle light scattering (MALS), quasi-elastic light scattering (QELS) and refractometer detector. MALS experiments were performed using an 18-angle DAWN HELEOS II light scattering detector connected in tandem to an Optilab refractive index concentration detector (Wyatt Technology). System normalization and calibration were performed with bovine serum albumin using a 45 μl sample at 10 mg/ml in the same SEC running buffer and a d*n*/d*c* value of 0.19. The light scattering experiments were used to perform analytical scale chromatographic separations for MW determination of the principle peaks in the SEC analysis. UV, MALS and differential refractive index data were analyzed using Wyatt Astra 7 software to monitor the homogeneity of the sample across the elution peak complimentary to the above-mentioned SEC-SAXS signal validation.

### Solution structure modeling

A pool of Ku conformers from our previous study (71) was used to fit the experimental SAXS profile of Ku using FOXS (49–51) and a single state model was selected using MultiFOXS (50) (Figure 3E and Supplementary Figure S12). The same pool of conformers plus KU/SL2 models, built based on the KU/ TLC1 complex (5y58.pdb, (52)), was used to fit experimental SAXS of KU5D+SL2 and KU5A+SL2. Two state model was selected by MultiFOXS (50) (Supplementary Figure S12). The experimental SAXS profiles were then matched to the theoretical scattering curves generated from atomistic models using FOXS (49–51). Minimal molecular dynamics (MD) simulations were performed on flexible regions in the crystal structure of PAXX (14) by the rigid body modeling strategy BILBOMD in order to explore conformational space (53). One state model was selected using MultiFOXS (50) (Figure 4 and Supplementary Figure S12); SAXS *ab initio* shaping using DAMMIF (54) followed by averaging DAMMAVER (55) was performed to obtain the average SAXS envelope of the Ku5A dimer. An atomistic model of Ku dimer was built based on the Ku–Ku interface found in crystal structure (1jeq.pdb, (56)), and was superimposed with the SAXS envelope (Figure 3G). This atomistic model, including the position of the Ku80CTR region as determined above were matched to the experimental SAXS of Ku5A dimer (Supplementary Figure S12D).

### DNA-PK kinase assays

2 pmole purified DNA-PKcs and Ku70/80 were incubated with 10 pmole purified XLF in 25mM Hepes-KOH pH 7.5, 50 mM NaCl, 10 mM MgCl_2_, 0.2 mM EGTA, 1 mM DTT, 2 pmole BSA and 2.5 mM ATP (added last) in a final volume of 20 μl. Samples were incubated at 30°C for 10 min then reactions were stopped by addition of 5× SDS sample buffer, boiled and analyzed on SDS PAGE followed by immunoblot with antibodies for DNA-PKcs (generated in house), Ku80 (Abcam 3107), XLF and XLF phospho-S245 (described in (57), as indicated.

### RNA interference (RNAi)

MDA-231 cells from ATCC (HTB-26) were cultured in RPMI-1640 media. The scrambled siRNAs (Sense strand: 5′-UUGCUAAGCGUCGGUCAAUTT-3′; Antisense strand: 5′-AUUGACCGACGCUUAGCAATT-3′), ON-TARGETplus SMARTpool siRNA targeting PAXX (L-026038, GE Healthcare Dharmacon) and XRCC5 (L-010491, GE Healthcare Dharmacon) siRNAs were prepared for transfection according to manufacturer’s protocols. Briefly, siRNAs were dissolved in opti-MEM at 50 μM using lipofectamine 3000 reagent (Thermo Fisher Scientific, USA), followed by 15-min incubation, and then they were added to 2 × 10^6^ cells in opiti-MEM media (final concentration = 50 nM). After 6  h, cells were washed with phosphate-buffered saline (PBS) and cultured in completed DMEM medium with 10% fetal bovine serum, and the cells were cultured for another 48 h before harvesting for qPCR or for RNA-FISH experiments.

### RNA-FISH using RNAScope**^®^** assay

Detection of *LINP1* expression using RNAScope^®^ probe (RNAscope® Probe - Hs-LINP1 (Cat #542071) is a 17 ZZ probe targeting the region 2 - 861 of human lncRNA in non-homologous end-joining pathway 1, designed by Advanced Cell Diagnostics.) was performed on MDA-231 breast cancer cell lines with RNAScope^®^ 2.5 High Definition Assay kit according to the manufacturer’s instructions (Advanced Cell Diagnostics) 45 min after irradiation with X-rays as indicated. The images were visualized with Zeiss Axioskop2 plus Microscope, and the slides were scanned on the Automated Cellular Image System III (ACIS III, Dako, Denmark) for quantification by digital image analysis.

### qRT-PCR

Total RNA was isolated from MDA-231 cancer cell lines using Trizol (Invitrogen) and digested with Turbo DNase (Ambion) to remove genomic DNA, according to the manufacturer’s instructions. The sequence of primers used for cDNA synthesis was PAXX Forward: 5′-AGCTTTGGAGCACCTGCTTCAC-3′, PAXX Reverse: 5′-AGAGTCAGAGCCACAGCTTGCT-3′, XRCC5 Forward: 5′-GTTCTAAAGGTCTTTGCAGCAAGA-3′, XRCC5 Reverse: 5′- AAAAGCCACGCCGACTTGAGGA-3′, ACTIN Forward: 5′- CACCATTGGCAATGAGCGGTTC-3′ and ACTIN Reverse: 5′- AGGTCTTTGCGGATGTCCACGT-3′. The cDNA was prepared by using iScript cDNA Synthesis Kit (Bio-Rad) and SYBR Green Supermix (Bio-Rad), respectively. Real-time PCR was run by using Bio-Rad CFX Connect Real-time System.

### Cryo-EM preparation

The LINP1 RNA samples were prepared for cryo-electron microscopy studies at the Baylor College of Medicine Cryo-Electron Microscopy Core Facility (BCM, Houston, TX). The grids were pretreated with a 30 s air-glow discharge immediately before vitrification. During vitrification, 3μl of 0.05 mg/ml RNA sample was applied to a freshly glow discharged grid (Quantifoil R2/1, Cu 200 mesh), blotted for 3 s and subsequently plunged into liquid ethane using a Vitrobot Mark IV (FEI Company, Hillsboro, OR) set at 4°C and 100% humidity. A JEOL2200FS (JEOL) electron microscope, fitted with a post-column energy filter set to 30 eV, was used for acquiring images of the vitrified RNA sample. Prior to imaging, the microscope was carefully aligned in order to minimize the effects of hysteresis and astigmatism. Images were collected at a magnification of 40 000× with a respective pixel size of 1.64 Å using a DE-64 camera (Direct Electron, San Diego, CA). Imaging was done with a dose rate of ∼30e^−^/Å^2^/s using a 1-s exposure time with a 24 frame/s capture rate. The final images used were automatically gain and dark corrected through the DE Data Collection (Direct Electron, San Diego, CA) software used to acquire said images.

### Negative staining preparation and imaging

The samples were prepared for negative stain electron microscopy studies at the Baylor College of Medicine Cryo-Electron Microscopy Core Facility (BCM, Houston, TX). Pre-made 2% uranyl acetate (UA) solution (EMS, Hatfield, PA) was filtered twice using a 0.2 μm filter attached to a 10 ml syringe. The grids (Quantifoil R2/1 + 2nm C, Cu 200 mesh) were pretreated with a 15 s air-glow discharge at 30 mA using a PELCO easiGlow™ Glow Discharge Cleaning System (Ted Pella, Inc., Redding, CA), making a hydrophilic surface. The negative stained grids were prepared using published ‘Side Blot Method’ protocol (58). A 3 μl drop of sample was applied to the carbon side of a freshly glow-discharged grid. After a 3-min incubation period, the excess buffer was removed, and the grid was washed with two 3 μl drops of deionized water. The grid was then stained with two 3 μl drops of UA. The first drop was incubated on the grid for 10 s and the second for 60 s.

A JEOL2100 (JEOL) electron microscope was used for acquiring images of the negatively stained sample. This instrument was equipped with an LaB_6_ filament and operated at an acceleration voltage of 200 kV. Images were collected at magnifications of 15 000× and 30 000× with respective pixel sizes of 3.43 and 1.59 angstroms using a DE-12 camera. Images were captured in the integrating mode with a dose rate of ∼36 e^−^/Å^2^/s and a 1 s exposure time with a 24 frame/s frame rate. The final images used were automatically gain and dark corrected through the DE Data Collection (Direct Electron, San Diego, CA) software used to acquire said images.

### Single-molecule studies using molecular forceps

#### DNA constructs

T-shaped primer oligonucleotides were coupled using an Azido-DBCO click reaction as previously described (38). A DNA PCR product of ∼3 kbp was amplified using the T-shaped oligos as primers and the Charomid 9–5 ΔSbfI as template, and purified by agarose gel electrophoresis (Macherey-Nagel). The PCR template-unmatched single strand of the T-shaped primers, Hyb1 and Hyb2 were annealed to their complimentary ssDNA oligos O1-Comp and O2-Comp, to form an AscI overhang (isocaudomer of MluI) and NsiI overhang (isocaudomer of SbfI), respectively. We denoted the annealed DNA products as ‘break’ DNA given the fact that it contains ‘free’ DNA ends accessible to NHEJ components and which could be digested by SmaI/XmaI restriction enzyme.

DBCO-Hyb1 5′-(dT)CCATGGGCATACTGATCGGTAGGG-3'

DBCO-Hyb2 5′-(dT)GAGCCAAGACGCCTCCATCCATGCA-3'

Az-Charomid-1456-SmaI (SmaI/XmaI restriction site is underlined):

5′-GAGAGACCCGGGCACCGTCTCCTTCGAACTTATTCGCAATGGAGTGTCATTCATCAAGGACGCCGC(AmC6-T/Azido) ATCGCAAATGGTGCTATCC-3'

Az-Charomid-3778-SmaI:

5′-GAGAGACCCGGGCACGACTTATCGCCACTGGCAGCAGCCACTGGTAAAGGATTAGCAGAGCGAGG(AmC6-T/Azido) ATGTAGGCGGTGCTACAGAG-3'

O1-Comp 5′-pho-CGCGCCCTACCGATCAGTATGCCCATGGA-3'

O2-Comp 5′-pho-TGGATGGAGGCGTCTTGGCTCA-3'

1x SureCut buffer (New England Biolabs)

The ∼680 bp ‘bridge/leash’ DNAs were obtained by PCR amplifcation of Charomid C9–5 ΔSbfI template with oligos Charo-3600-MluI and Charo-4230-SbfI, and the ∼6000 bp ‘bridge/leash’ DNAs were obtained by PCR amplifcation of λ-DNA template with oligos λ-40001-MluI and λ-46000-SbfI.

Charo-3600-MluI 5′-GAGAGAACGCGTTACCTGTCCGCCTTTCTCCCTTCGGG-3'

Charo-4230-SbfI 5′-GAGAGACCTGCAGGC CTCACTGATTAAGCATTGGTAACTGTCAGACC-3'

λ-40001-MluI 5′-GAGAGAACGCGTTCCGGATGCGGAGTCTTATCCGTGGAAATC-3'

λ-46000-SbfI 5′-GAGAGACCTGCAGGACCAGAGCGGAGATAATCGCGGTGACTCTG-3'

The aforementioned ‘break’ DNA and ‘bridge’ DNAs were treated first by a mix of SbfI, MluI-HF, NsiI, AscI, HC-T4 DNA ligase, and then by a mix of XbaI and SacI (New England Biolabs) to generate DNA forceps with symmetric backbone 1.5 kbp × 2 linked by ∼680 or ∼6000 bp leashes. These DNA forceps contain XbaI and SacI overhangs, allowing ligation to biotin-labeled ∼1 kbp DNA bearing an XbaI site and digoxigenin-labelled ∼1 kbp DNA bearing a SacI site for attachment in the magnetic trap to, respectively, streptavidin-coated magnetic bead and antidigoxigenin-modified glass surface. These DNA forceps also contain SmaI/XmaI restriction sites allowing us to generate blunt or sticky DNA ends mimicking the DNA double breaks. Detailed enzyme concentrations and reaction time were as previously described (38).

For assembly onto the microscope, the ligation reaction was first diluted 30-fold to 100 pM nominal concentration of DNA in Tris buffer (10 mM Tris-Cl pH 8.0). Then, 0.5 μl of this dilution was mixed with 10 μl of Dynal MyOne C1 magnetic beads. The beads were prepared by taking 10 μl of stock solution, washing them with 200 μl of RB and concentrating the beads, discarding the supernatant and resuspending the beads in 30 μl of RB). The beads + DNA mixture was incubated at room temperature for 3 min before injection onto anti-digoxigenin functionalized surfaces. After incubating at room temperature for 10 min, the bead + DNA assemblies not bound to the surface were washed away gently using 5 × 200 μl of RB. Typically, ∼30–50 functional DNA molecules tethered in one field of view (120 μm x 140 μm) could be monitored simultaneously.

#### Proteins for single-molecule studies

The expression and purification of Ku70/Ku80, DNA-PKcs, PAXX, full-length LigaseIV-XRCC4 was performed as previously described (14,38,59).

#### Experimental conditions

All single-molecule assays were conducted at 34°C in reaction buffer RB (20 mM K•Hepes pH 7.8, 100 mM KCl, 5 mM MgCl_2_, 1 mM ATP, 1 mM DTT, 0.05% Tween-20, 0.5 mg/ml BSA). When present, NHEJ components were used at a concentration of 1 nM DNA-PKcs, 1 nM Ku70/80 (heterodimer), 10 nM PAXX (monomer), 10 nM XLF (monomer) and 10 nM XRCC4 -Ligase IV. Short LINP1 fragments SL2 was used at 50 μM, and full-length LINP1 (1–917 nt) at 10 nM, with 0.1 U/μl RNaseOUT inhibitor (Invitrogen). To test the ligation of DNA molecules, 0.1 U/μl of T4 DNA ligase was added, after which the ligated DNA was cleaved using either 0.2 U/μl XmaI or 0.1 U/μl of SmaI (New England Biolabs).

#### Data collection and analysis

Single-molecule magnetic trapping data giving DNA extension as a function of time and under automatic force-modulation cycles were obtained using the Picotwist software suite (Picotwist S.A.R.L). Magnetic trapping was carried out as described previously (38). A typical modulation cycle was ∼120 s at 0.01 pN with ∼120 s at a 1.4 pN pulling force, except for experiments carried out with T4 DNA ligase with a cycle of ∼300 s at 0.01 pN and ∼100 s at 1.4 pN. Time-traces were analyzed to extract the fraction of traction cycles for which, upon application of the higher pulling force, the DNA extension is initially observed to be shorter than the maximum extension, but then recovers maximum extension during the course of that same high-force portion of the traction cycle (referred to as frequency of end-binding events). We also extracted all amplitudes of end-binding events ruptured during the high-force portion of the traction cycle. Finally, for all those end-binding events in which end-binding ruptured within the same traction cycle, we determined the duration of the end-binding event. We only analyze the lifetime of end-binding events for which the change in DNA extension observed upon rupture is consistent with specific synapsis (i.e. within three standard deviations of the expected length of amplitude change at the higher pulling force).

## RESULTS

### LINP1 is a flexible non-coding RNA that can self-assemble into phase-separated droplets

**Figure 1.**
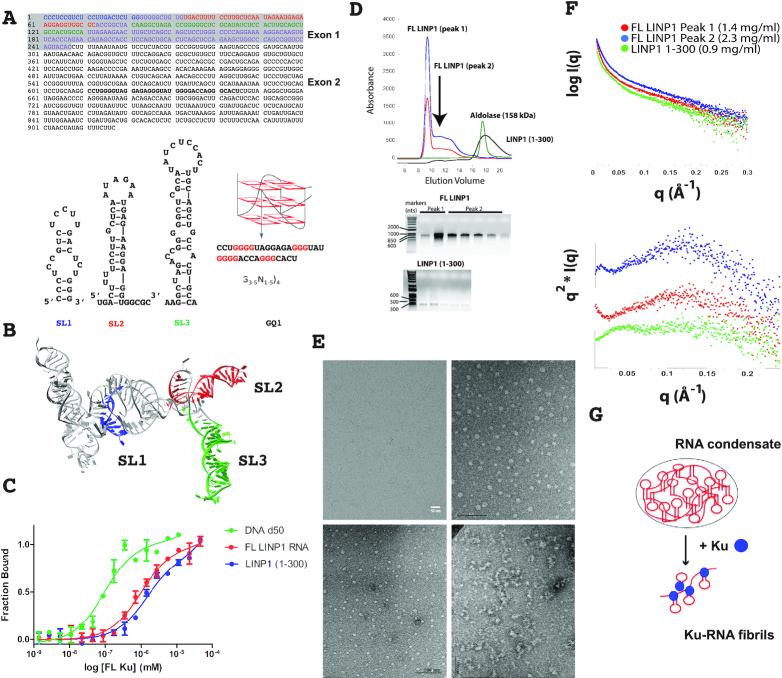
LINP1 sequence, structure and assembly. (**A**) Sequence of LINP1 RNA. Oligonucleotide SL1 (nt 1–22) is colored in blue, SL2 (nt 33–72) in red, SL3 (nt 81–130) in green and GQ1 (nt 611–645) in black and underlined. The region of LINP1 suggested to interact with Ku (30) is shown in grey. Computationally predicted secondary structures that were confirmed by NMR and biophysical analyses (this study) are shown for SL1 (B), SL2 (C), SL3 (D) and the predicted G-quadruplex motif. (**B**) A tertiary structure model of LINP1 (1–300) constructed with RNAComposer using the secondary structure elements defined from NMR analysis for the three stem-loops in this region. (**C**) Binding curves for MST data for the interaction between FL Ku and d50 DNA (in blue), FL LINP1 (in red) and LINP1 (1–300) (in green). FL Ku binds DNA with 40–100 fold higher affinity than LINP1 RNAs (Supplementary Table S3). (**D**) Purification of FL LINP1 and LINP1 (1–300). *In vitro* transcribed FL LINP1 and LINP1 (1–300) purified by SEC. For FL LINP1, the A260 is shown in blue and the A280 in red. The RNA elutes close to the void volume in two peaks (Peaks 1 and 2). At low concentrations, LINP1 (1–300) (*M*_r_ ∼ 90kDa) eluted in a broad peak, close to that expected for aldolase (158 kDa). (**E**) Cryo-EM and negative stain EM images of FL LINP1 and LINP1 (1–300). Clockwise from top left: Cryo-EM image of FL LINP1 at 0.05 mg/ml, negative stain image of FL LINP1 at 0.05 mg/ml in the presence of 5% PEG400, negative stain image of FL LINP1-Ku complex at 0.1 mg/ml, negative stain image of LINP1 (1–300) at 0.1 mg/ml. (**F**) SAXS analysis of LINP1 RNA samples. (top) SAXS curves for FL LINP1 peak 1 from SEC (in red), peak 2 from SEC (in blue), and LINP1 (1–300) (in green). (Bottom) Dimensionless Kratky plots indicate the level of disorder. (**G**) A plausible model for Ku-LINP1 ribonucleoprotein complex (derived from the EM data) that results in dissociation of the RNA droplets. The model predicts that several Ku70/80 heterodimer complexes likely recognize a single LINP1 molecule.

To study LINP1 structure and flexibility and probe its interaction with Ku, we transcribed the full-length (FL) 917 nt RNA and nts 1–300 (Figure 1A and B) and tested various folding conditions, with and without heat denaturation, as well as a range of Mg^2+^ concentrations (0–50 mM). The full-length RNA was purified to homogeneity using SEC (Figure 1D) and tested for function, i.e. its ability to bind Ku, by microscale thermophoresis (MST) (Figure 1C), as well as in a single-molecule system to test for promoting synapsis (described below). Our measured affinity for DNA is consistent with that reported in the literature for a number of DNA substrates (Supplementary Table S1). The purified Ku heterodimer binds DNA with an apparent equilibrium dissociation constant (*K*_d_^app^) of ∼19 nM, 40× tighter than the binding of Ku to FL LINP1 (*K*_d_^app^ ∼740 nM) (Supplementary Tables S1 and S3). The Hill coefficient was 1.75 for the 50 bp DNA, indicative of cooperative binding of two Ku molecules on a 50 bp DNA, and was 1.0 for both FL LINP1 and LINP1 (1–300) indicating no cooperativity for Ku binding to RNA. Therefore, Ku binding to LINP1 RNA is weaker than its association with DNA.

The FL LINP1 RNA eluted in two peaks by SEC, one close to the void volume (Peak 1, Figure 1D) was an aggregate and less active in the single-molecule synapsis assay, and a second peak that also eluted as a larger-than expected species (*M*_r_ ∼ 300 kDa, Peak 2, Figure 1D), but was functionally active in its ability to facilitate synapsis. No LINP1 RNA was present in fractions corresponding to the expected molecular weight. The SEC elution profile for LINP1 nts 1–300 (Figure 1D) was broad and eluted closer to 158 kDa (expected *M*_r_ ∼90 kDa) for the bulk of the RNA transcribed, although a significant fraction also eluted close to the void volume.

To characterize the structural and dynamic properties of these RNAs, we employed SAXS analysis (48) (Figure 1F) and cryo-electron microscopy (cryo-EM) imaging at a range of concentrations (Figures 1E and Supplementary Figure S3). SAXS is uniquely suited to study the conformational dynamics and architectures of macromolecules such as lncRNAs, which cannot crystallize and are too large to be studied by NMR (60,61). At concentrations ranging from 0.9 to 2.3 mg/ml, all RNAs showed nonlinearity in the low-*q* range (the Guinier (62) region) of the SAXS curves (Figure 1F) such that *q* × *R*_g_ was > 1.3, which is indicative of aggregation (45). Kratky–Debye plots of the SAXS scattering curve (Figure 1F) can provide information about the degree of compactness in the RNA (63). A Gaussian distribution with *q*-max (Å) approaching zero is observed for compact particles; however, the LINP1 RNAs SAXS curves did not show a clear Gaussian profile, but plateaued at high *q* with a Porod exponent < 3 (Supplementary Table S4), indicating high flexibility. The SAXS data were further corroborated by cryo-EM images collected at 0.05 mg/ml that verified that at low concentrations, full-length LINP1 is not folded into a compact tertiary structure, but remains flexible and dynamic in solution (Figure 1F). We collected negative stain EM images of LINP1 (1–300) at a range of concentrations under physiological buffer conditions. At 0.1 mg/ml, LINP (1–300) existed as in equilibrium between a flexible particle and a phase-separated condensate or droplet. These RNA droplets ranging in size from 10 to 17 nm were very apparent between 0.3 and 0.5 mg/ml (Figure 1E and Supplementary Figure S3), and no droplets were observed in the buffer control. Very similar droplets ranging in size between 10 and 24 nm were observed for the full-length LINP1 in the presence of 5% PEG400, a common reagent used to mimic the crowding conditions of the cell (Figure 1E and Supplementary Figure S3). These droplets were too small to be observed by light microscopy using Cy3-labeled LINP1 (data not shown). These data suggest that LINP1 RNA exists at equilibrium between a flexible monomeric structure and a phase-separated granule that is formed via RNA–RNA interactions alone. RNA flexibility and the presence of a G-quadruplex in LINP1 (see below) likely promote formation of higher order oligomers. Addition of Ku to full-length RNA or LINP1 (1–300) resulted in a loss of RNA granules by negative stain EM and formation of long fibers suggesting a filamentous assembly of the Ku–RNA complex (Figure 1G). SEC-SAXS and MALS of the Ku-LINP1 samples suggests oligomerization of Ku on LINP1 as the samples eluted in the void volume (Supplementary Table S4).

### RNA flexibility and a G-quadruplex in LINP1 lncRNA drives RNA–RNA interactions

To determine whether Ku70/80 interacts with specific secondary structures, we first used Mfold (http://unafold.rna.albany.edu/?q=mfold) to predict secondary structures in the LINP1 RNA (Figures 1B; Supplementary Figure S4 and Supplementary Table S2). This computational analysis suggested that nts 1–130 in the LINP1 5′ end could potentially form three stem-loops, which we designated SL1, SL2 and SL3. In addition, we identified a region in the middle of the LINP1 sequence between nts 611–645 (Figure 1A and Supplementary Table S2) that had a consensus sequence for a G-quadruplex or G4 motif (G_3–5_N_1–5_)_4–6_. Whereas Ku has previously been reported to bind RNA hairpins (16–18,64,65), RNA G-quadruplexes have not previously been shown to be a substrate for the Ku heterodimer.

To experimentally test whether the LINP1 stem-loops and G4 motif formed *in vitro*, the sequences (Supplementary Table S2) were characterized biophysically. Circular dichroism (CD) spectroscopy showed that SL1, SL2 and SL3 stem-loops all had characteristic CD spectra with *λ*_max_ and *λ*_min_ at 262 and 209 nm, respectively, consistent with formation of hairpin RNA structures (Supplementary Figure S4) whereas the G-quadruplex showed a *λ*_max_ at 260 nm and a *λ*_min_ at 240 nm, confirming formation of a G4 structure only in the presence of potassium (but not lithium). To determine whether the LINP1 stem-loops showed Watson–Crick (W-C) base pairing as predicted by computation, we collected imino proton NMR spectra (Supplementary Figure S5). The NMR data also indicate that stem-loop structures form with the expected W-C base pairs (Supplementary Figure S5). In contrast to the stem-loops, the G-quadruplex motif showed extreme broadening of imino resonances, indicating that this domain had a propensity to form higher order structures, and may in part be responsible for the aggregation observed in full-length LINP1 RNA.

**Figure 2.**
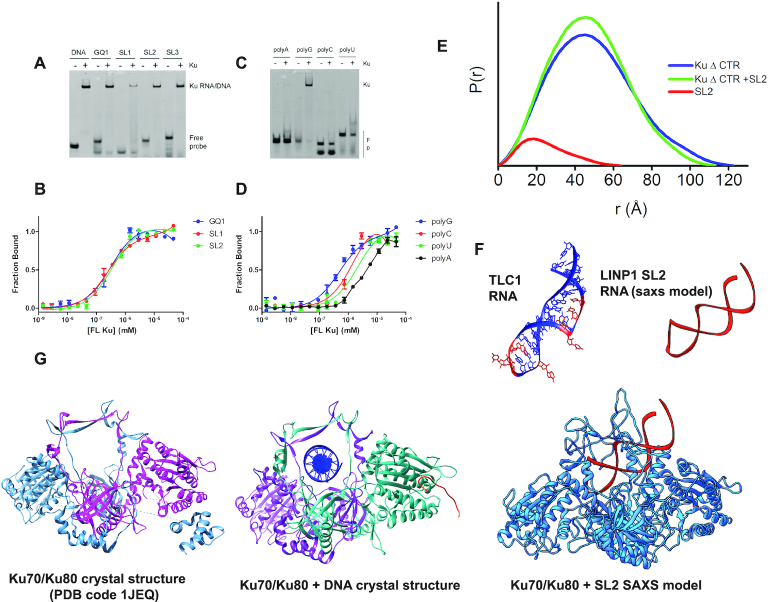
LINP1 stem-loops and G-quadruplex RNAs bind Ku. (**A**) Ku70/80 was purified from HeLa cells (Ku) or expressed from baculovirus (bKu) as either full-length Ku70/Ku80 (FL), Ku70/Ku80 (1–718) and 4 pmole was incubated with either 2 pmole of the structured RNA substrates GQ1 RNA, 25bp DNA, SL2 RNA or SL3 RNA and analyzed by EMSA. (**B**) The interaction of FL Ku with structured RNA substrates was examined by MST. The *K*_d_^app^ values are in Supplementary Table S3. (**C**) A similar interaction of FL Ku with single-stranded substrates polyA, polyG, polyC and polyU is shown using EMSA and the MST curves are depicted in panel (**D**). (**E**) Normalized *P*(*r*) functions calculated from the SEC-SAXS data are shown for Ku ΔCTR (1–569), Ku-ΔCTR-SL2 complex and SL2 alone. (**F**) Crystal structure of the TLC1 RNA bound to Ku (pdb code 5y58) and the SAXS derived model for SL2 LINP1 RNA (shown in red). (**G**) Ensemble models of Ku-SL2 calculated using the solution SAXS data and BILBOMD is shown (far right). The Ku heterodimer is in blue and the SL2 RNA model in red. This SAXS derived model is compared with the crystal structures of the Ku heterodimer free (PDB code 1JEQ) and Ku70 (in purple)/Ku80 (in green) heterodimer bound to DNA (shown in blue) and APLF peptide (shown in orange) (left).

The oligomeric states of SL1, SL2 and SL3 and the G-quadruplex were determined using SEC-MALS (Supplementary Figure S7) as well as SAXS (Supplementary Table S4 and Supplementary Figure S7). The SL1, SL2 and SL3 stem-loops were predominantly monomeric by SEC-MALS with a molar mass close to the expected molecular weights. In contrast, the G-quadruplex showed a broad elution profile (Supplementary Figure S7), extending from a monomer with a molar mass of 13.6 kDa to a mass of 262 kDa, with several species between 13.6 and 262 kDa. SAXS analysis of LINP1 RNA sub-domains also indicated that the stem-loops were structured and well-ordered in solution whereas the GQ1 motif formed a large extended structure with a linear radius of gyration (*R*_g_) of 85 Å and a maximal dimension (*D*_max_) of 245 Å (Supplementary Figure S7). Calculation of independent models of SL2 RNA from the SAXS data was consistent with formation of a stem-loop structure (Figure 2F). The *R*_g_ values and molecular weights determined for the stem-loops from SAXS were in agreement with the SEC-MALS data. The normalized Kratky–Debye plots (Supplementary Figure S7) for the stem-loops indicated fairly compact structures with the Porod exponent ranging from 2.9 to 3.4 (Supplementary Table S4); however, the Kratky–Debye plot for the G-quadruplex showed a plateau that did not approach baseline at high *q*, clearly suggestive of a flexible particle, similar to that observed for the full-length LINP1 RNA. Our data suggest that RNA secondary structure, and in particular G-quadruplexes may be a major driver for formation of RNA–RNA aggregates.

### Ku binds structured RNAs in a manner similar to the TLC1 RNA stem-loop

To determine whether Ku prefers specific RNA substrates, we tested representative single-stranded (ss) and structured RNAs for Ku binding. Ku bound all three LINP1 SL1, SL2 and SL3 RNAs in an EMSA assay (Figure 2A). MST analysis using FAM-labeled RNAs showed that the *K*_d_^app^ of full-length Ku heterodimer toward SL1, SL2 and SL3 substrates (Figure 2 and Supplementary Table S3) was between 0.84 and 1.69 μM. Intriguingly, although all three stem-loops vary in the length of the stem and the size of the loop, they have one or two bulges in the stem region. To determine whether the bulge is necessary to Ku binding, as predicted previously (16), we tested binding of Ku to RNA substrates with base transversions in the predicted stem region. A 6× decrease in affinity was observed when all of the base pairs in the stem were replaced by their Watson–Crick complement for SL2 RNA and a 2× decrease for SL3 (Supplementary Table S3, Supplementary Figures S6 and S9C). Replacement of the loop sequences in SL2 and SL3 hairpins by uridines also decreased binding toward Ku by about 2-fold. We also tested whether Ku could recognize a stem-loop conserved in the 3′ untranslated region of histone mRNAs (66) that lacks a bulge. This 26 nt sequence from histone H3 has been structurally characterized by NMR (67–69) (Supplementary Table S2). No binding was observed to the histone H3 stem-loop by EMSA (Supplementary Figure S8). These data are in line with those reported for TLC1 RNA, where a bulge in the RNA stem is required for Ku recognition (16). Consistent with the 40–100× higher affinity of Ku for DNA over RNA (Supplementary Table S3), we were not able to compete RNA binding of the stem-loops with a pre-formed Ku–DNA complex (Supplementary Figure S8B).

Intriguingly, eCLIP data deposited in the ENCODE database (Supplementary Figure S10) show that Ku is bound to a wide range of RNA targets in HepG2 and K562 cells. In HepG2 cells, ∼72% of Ku targets were the distal or proximal introns of mRNAs indicating Ku may play an as yet unidentified role in RNA splicing. Moreover ∼17% of Ku targets were ncRNAs that include lncRNAs such as NEAT1, snoRNAs, snRNAs and vaultRNAs in HepG2 cells. GO analysis indicates that in HepG2 cells Ku is localized to nuclear speckles, consistent with its putative role in RNA splicing. Similarly, in the leukemia cell line K562 (Supplementary Figure S10), ∼47% of Ku targets were proximal and distal introns of mRNAs, ∼20% corresponded to the coding region of mRNAs, ∼15% to non-coding RNAs, ∼3% to tRNAs and <1% to miRNAs. GO analysis in K562 cells also suggests Ku is likely involved in RNA splicing reactions, RNA Pol II transcription activity, rRNA processing and translation. In both HepG2 and K562 cells, motif analysis showed that Ku did not bind a specific RNA sequence. Collectively, our study, along with publicly available eCLIP data, reveals that Ku is a legitimate RNA-binding protein that has dual roles in both RNA and DNA metabolism.

**Figure 3.**
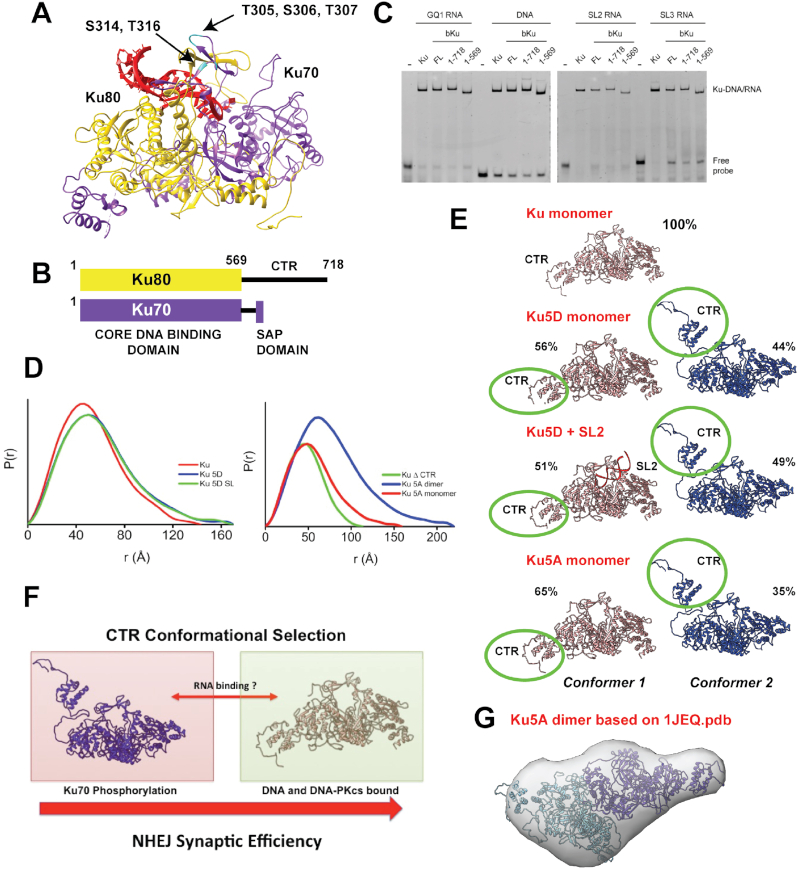
Multi-site phosphorylation of Ku70 at five sites and LINP1 SL2 RNA regulates orientation of Ku80 CTR that is unnecessary for RNA binding. (**A**) Crystal structure of the Ku heterodimer showing the sites of Ser/Thr phosphorylation in the central β-channel. (**B**) Schematic domain structure of Ku70 and Ku80. (**C**) Ku70/80 was purified from HeLa cells (Ku) or expressed from baculovirus as either full-length Ku70/Ku80 (FL), Ku70/Ku80 (1–718) or Ku70/Ku80 (1–569) as indicated and 4 pmole was incubated with 2 pmole GQ1 RNA, 25bp DNA, SL2 RNA or SL3 RNA and analysed by EMSA. (**D**) Normalized *P*(*r*) functions calculated from the SEC-SAXS data are shown for Ku WT or mutant proteins +/− SL2 RNA. A comparison of the normalized P(r) functions for FL Ku, Ku ΔCTR (Ku70/Ku80 (1–569)), Ku5A and Ku5D is shown. (**E**) Ensemble conformers of Ku proteins +/− SL2 RNA as described in the text. (**F**) Our proposed model for NHEJ synaptic efficiency that is dependent on RNA and the phosphorylation state of the central β-barrel. The model suggests that NHEJ synaptic efficiency is directly correlated with the orientation of the Ku80CTR. (**G**) The dimer SAXS envelope derived from the Ku5A data from DAMMIF superimposed on the structure of the crystallographic Ku dimer (PDB code: 1JEQ).

To understand how Ku binds LINP1 RNAs we purified full-length Ku (i.e. Ku70 plus Ku80 (1–718) or a complex lacking the extreme C terminal region (CTR) of Ku80 (Ku ΔCTR, residues 1–569) (Figure 3B). The Ku80 CTR forms a flexible arm that interacts with DNA-PKcs, recruiting it to DNA bound Ku (41,70,71). Both proteins were well behaved and monodisperse by SEC-SAXS and MALS (between 3 and 10 mg/ml). We determined the ability of Ku ΔCTR to interact with GQ1, SL2 and SL3 RNAs using EMSA, MST and SEC-SAXS (Figure 3C and D). Deletion of the Ku80CTR had no effect on the interaction of Ku with DNA or RNA by EMSA, suggesting that the RNA interacting region is within the central DNA binding region of Ku70/80 or the Ku70 SAP domain. This was confirmed by MST titrations that yielded very similar *K*_d_^app^ for both Ku heterodimers (Supplementary Table S3). As previously shown (71), comparison of SAXS parameters for FL Ku and Ku ΔCTR (Supplementary Table S4) revealed that the C-terminal 150 amino acids in Ku80 are flexible with *R*_g_ values of 43 and 38 Å and a larger *D*_max_ for FL Ku compared to Ku ΔCTR (150 Å versus 120 Å, Figure 3D). A decrease in the Porod exponent (*P*_x_) to 3.2 from 3.6 for FL Ku70/80 and Ku80 ΔCTR, respectively, is also consistent with the flexibility of the CTR.

To examine the solution structure of the Ku-SL2 RNA complex, we determined the experimental pair distribution function *P*(*r*) for FL Ku and the KuΔCTR mutant in the presence and absence of SL2 (Figure 2E; Supplementary Figure S12 and Supplementary Table S4). Binding of SL2 to the Ku ΔCTR leads to a narrowing of the pair distribution function *P*(*r*) and supports SL2 binding within the central β-barrel between Ku70 and Ku80, and agrees with our previous SAXS observation of Ku–DNA complexes (71) (Figure 2E). A similar but smaller narrowing of the *P*(*r*) functions was observed for FL Ku and FL Ku-SL2 (Supplementary Figure S12C). The reconstructed atomistic models of Ku ΔCTR-SL2 (see ‘Materials and Methods’ section) (Figure 2F and Supplementary Figure S12A) using the Ku-TLC1 RNA structure (52) as a template confirms the shape of Ku observed in the KU/TLC1 RNA complex structure and closely match the experimental SAXS profiles (Supplementary Figure S12A). Taken together, the results support our models derived from SAXS data (Figure 2F) as well as work in yeast on the Ku–TLC1 RNA complex (52), showing that the SL2 RNA is accommodated in the central β-barrel between Ku70 and Ku80.

Our discovery of a G-quadruplex in LINP1 RNA (GQ1) prompted us to examine whether Ku could bind G-quadruplex sequences. To our surprise, Ku bound GQ1 from LINP1 as well as the TERRA RNA G-quadruplex (72,73) in EMSA (Supplementary Figure S9A and S9B) and MST assays (Figure 2A and Supplementary Table S3) with an affinity higher than that for the stem-loops (*K*_d_^app^ between 110 and 430 nM). However, Ku did not recognize a DNA G-quadruplex of the same sequence, which likely has a different fold. Thus, Ku G-quadruplex binding is RNA specific (Supplementary Figure S8A). Ku recognition of RNA G-quadruplexes is further corroborated by our results on single-stranded RNAs. Whereas Ku bound weakly to polyA, polyC and polyU (Figure 2C and Supplementary Table S3), it bound polyG with a *K*_d_ of 170 nM. PolyG tracts are known to form G-quadruplexes and recognition of polyG by proteins such as the polycomb repressor complex PRC2 that bind RNA G-quadruplexes has recently been documented (74).

Although the list of RNAs that can bind Ku is increasing, none of the RNAs identified to date share a common sequence. Rather, it appears that Ku prefers structured RNAs like 25–48 nt RNA stem-loops with bulged out nucleotides as well as RNA G-quadruplexes. Therefore, Ku RNA binding specificity is likely dictated by the RNA shape and structure rather than sequence.

### Ku70 multi-site phosphorylation and RNA regulate the conformation of the Ku80 CTR

We previously reported (75) that multi-site phosphorylation of Ku70 at a cluster of five Ser/Thr residues (Thr305, Ser306, Thr307, Ser314 and Thr316) or mimicking phosphorylation at these sites by aspartic acids (Ku5D; Figure 3A) in the central β-barrel decreases its affinity for forked DNA substrates in EMSA assays. Ku dissociation from DSB ends by multi-site phosphorylation was necessary for initiation of DNA resection and triggering homologous recombination during S-phase (75). We confirmed this result in studies reported herein: the Ku5D mutant shows a 20-fold decrease in binding affinity (*K*_D_ ∼15 μM) toward a forked DNA substrate (Supplementary Figure S12E), although binding of WT FL Ku was also weaker toward the forked substrate compared to a d50 blunt-ended DNA substrate (Figure 1C). In contrast, the Ku5D mutant bound FL LINP1 RNA with 2-fold better affinity compared to the wild-type protein (Supplementary Figure S12E). This suggests that either the mode of RNA binding by Ku is different compared to DNA or phosphorylation at the central β-barrel results in a conformational change in Ku that has opposing effects on the RNA bound complex as compared to DNA.

To determine the mechanistic basis for this difference between RNA and DNA recognition, we collected scattering data for WT and mutant Ku proteins under the same buffer conditions. The *P*(*r*) functions for Ku5D and 5A mutants shows a profile skewed to the right consistent with a more extended/dynamic structure compared to the WT protein (Figure 3D and Supplementary Table S4). This increased flexibility could be due to the loss of stabilizing side-chain to main-chain hydrogen bonds observed for Ser 306, Thr 307, Ser 314 and Thr 316 in the Ku crystal structures (56) (Supplementary Figure S11). In addition, we used rigid-body modeling to compare the SAXS-derived models of WT FL Ku, FL Ku5A and FL Ku5D mutants, free and complexed to SL2 RNA (Figure 3E and Supplementary Figure S12). SAXS of FL WT Ku can be matched with a single conformation that juxtaposes the CTR domain against the core Ku DNA-binding domain (Figure 3E). Addition of SL2 RNA to FL WT Ku resulted in narrowing of the *P*(*r*) that indicates insertion of SL2 RNA in to the central β-barrel, and agrees with our SAXS studies of Ku–DNA complexes (71). The observed SAXS differences for FL Ku are smaller than differences observed for Ku ΔCTR upon SL2 complexation (Figure 2E) due to the smaller size of SL2 relative to the FL Ku and the presence of flexible protein regions in the FL Ku protein. However, the FL Ku-SL2 SAXS curve matched (Supplementary Figure S12B) the Ku atomistic model with SL2 located in central β-barrel quite well (Supplementary Figure S13A). SAXS profiles for the Ku5D mutant could be matched by two conformers where the CTR fluctuated between an ensemble of two major populations, only one of which resembles WT protein (∼56%), and a second minor conformer (∼44%) where the CTR was oriented away from the core DNA-binding region of the Ku heterodimer (Figure 3E). The Ku5A mutant also showed ensemble averaging between two major conformational species for the CTR, although the WT-like conformer was favored (65% (major) versus 35% (minor)). These data support a conformational selection model (Figure 3F) for NHEJ activation via modulation of the conformational state of the Ku80 CTR. We suggest that the major conformer we observe for the Ku80 CTR in the WT protein is one that is favored for interaction with DNA-PKcs, thereby increasing the efficiency of formation of the synaptic complex. The second minor conformer may be promoted by Ku70 phosphorylation or by mimicking phosphorylation by aspartic acids to disassemble the Ku–DNA–PK complex, allowing for initiation of DNA resection. RNA appeared to favor the second minor conformation, although the exact significance of this is unclear at this time.

### RNA induces Ku dimer-of-dimers

We consistently observed that a small but significant fraction of Ku elutes as a ‘dimer-of-dimers’ or a hetrotetramer in SEC-MALS (referred to as Ku dimer from here on) in the presence of SL2 RNA. These dimers are not due to non-specific aggregation of Ku as no dimers are observed in the absence of RNA. RNA-induced dimers were observed for both FL Ku as well as the Ku ΔCTR (data not shown), therefore dimerization is not mediated by the CTR. Intriguingly, the dimer population was greater in the Ku5D and Ku5A mutants (Figure 3D), which allowed us to model the Ku dimer from the SAXS data and the crystal structure (PDB code: 1jeq) (Figure 3G). The ability of Ku to form dimers has previously been reported in the presence of 24 bp DNA fragments (76) and was characterized by electron microscopy (EM) and atomic force microscopy (AFM) where end-to-end bridging of two DNA ends was facilitated by Ku multimers. Further evidence for Ku multimerization across the DNA bridge is also provided by pull-down experiments of ^32^P-labeled dsDNA using biotinylated DNA fragments as bait (77). Efficient pull-down of radiolabeled dsDNA was observed in the presence of streptavidin beads. Our data show that like DNA, RNA fragments can also support Ku multimerization and may help stabilize the initial synaptic complex.

### The Ku–LINP1 RNA interaction does not affect DNA-PK kinase activity

Human telomerase RNA hTR interacts with Ku and supports DNA-PKcs-dependent phosphorylation of heterogenous nuclear ribonucleotide protein hnRNPA1 (78). To determine whether LINP1 supported DNA-PK activity, we incubated purified DNA-PKcs and Ku70/80 with GQ1, SL1, SL2 or SL3 RNAs and assayed for DNA-PK activity toward XLF serine 245, a site we have shown previously is DNA-PK dependent *in vitro* and *in vivo* (57). No XLF phosphorylation was detected in this assay, while phosphorylation in the presence of 25bp dsDNA was robust (Supplementary Figure S14). Similarly, full-length LINP1 also did not support DNA-PK kinase activity *in vitro* (Supplementary Figure S14B) and addition of LINP1 RNA had no effect on DNA-dependent phosphorylation of XLF (Supplementary Figure S14C). Thus, LINP1 does not appear to support DNA-PK kinase activity, although we cannot exclude the possibility that LINP1 supports DNA-PKcs autophosphorylation (79), or phosphorylation of other DNA-PK substrates, perhaps in the context of RNA–protein complexes. These data are in agreement with our SAXS-derived model (Figure 3F) that suggests that RNA could orient the C-terminal region of Ku80 in a conformation unfavorable for interaction with DNA-PKcs.

### The Ku–LINP1 complex associates with XLF and APLF but competes with PAXX

**Figure 4.**
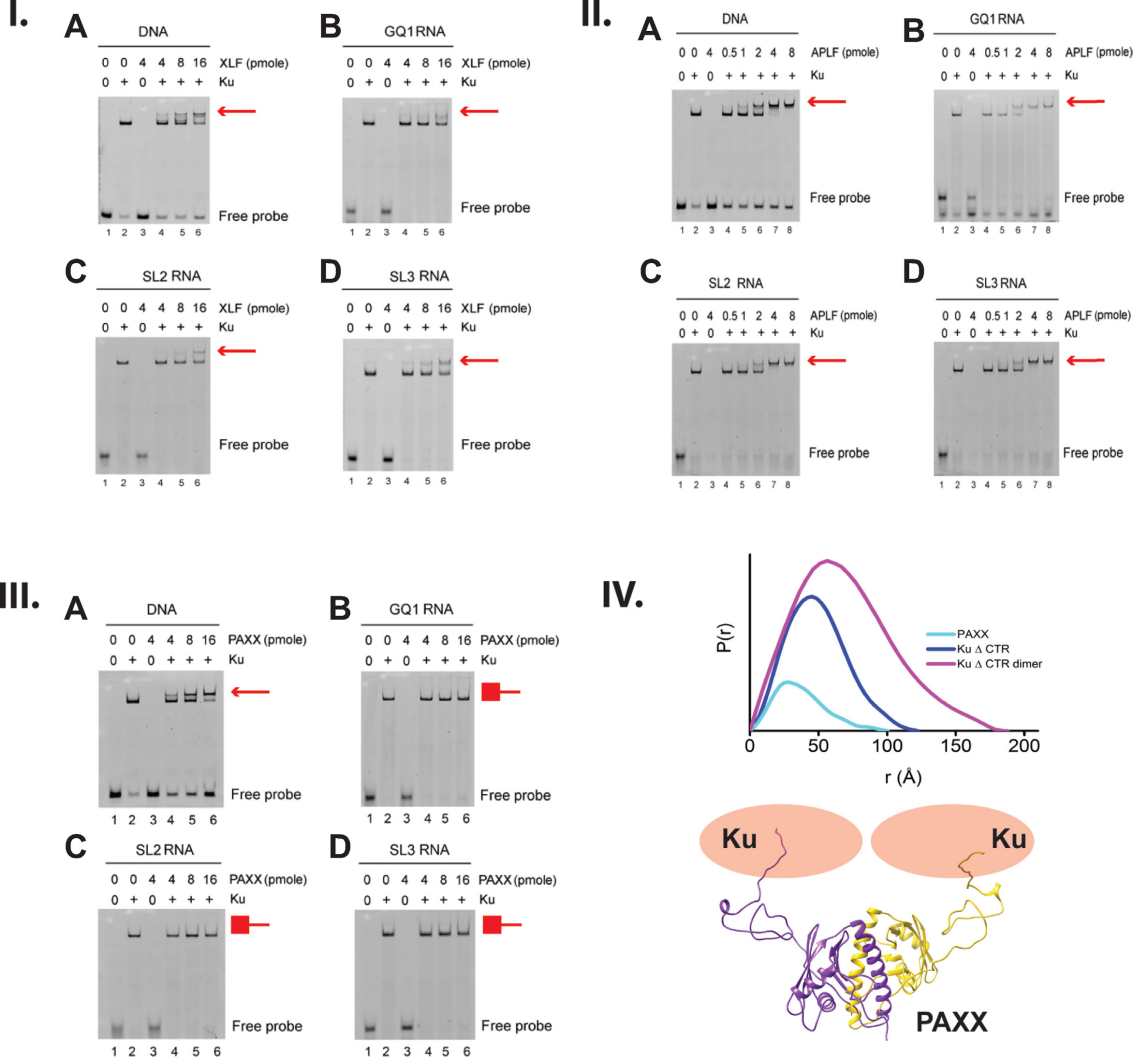
RNA binding facilitates higher order assembly of protein–RNA complexes to facilitate NHEJ. (I) *Ku and XLF interact in the presence of RNA*. 4 pmole Ku was incubated with 2 pmole 25 bp duplex DNA or 2 pmole GQ1, SL2 or SL3 RNA with 4–16 pmole XLF as indicated. (II) *Ku and APLF interact in the presence of RNA or DNA*. 4 pmole Ku was incubated with 2 pmole 25 bp duplex DNA or 2 pmole GQ1, SL2 or SL3 RNA with 0.5–8 pmole APLF as indicated. (III) *Ku and PAXX interact in the presence of DNA but not RNA*. 4 pmole Ku was incubated with 2 pmole 25 bp duplex DNA or 2 pmole GQ1, SL2 or SL3 RNA with 4–16 pmole PAXX as indicated. (IV) (Top) Normalized *P*(*r*) functions calculated from the SEC-SAXS data are shown for Ku-ΔCTR (1–569), Ku-ΔCTR dimer and PAXX alone. (Bottom) Our SAXS derived model for the PAXX heterodimer showing how each PAXX monomer could interact with Ku via Ku70.

We next examined whether LINP1 RNA supported interaction of Ku with other components of the NHEJ pathway, as observed for DNA. Full-length purified Ku was incubated with FAM-labeled dsDNA, GQ1, SL2 or SL3 RNA alone, or in the presence of increasing amounts of XLF, APLF or PAXX and samples were analyzed by EMSA as described. The addition of XLF super-shifted Ku–DNA complexes, consistent with interaction of Ku with XLF (12) (Figure 4l). Similar super-shifts were seen for Ku with DNA and APLF (80,81) and PAXX (82) (Figure 4I, II and III). Interestingly, super-shifted bands were also observed with Ku and XLF and Ku and XLF in the presence of GQ1, SL2 and SL3 RNA (Figure 4I and II) suggesting that Ku is able to interact with both RNA and XLF or APLF simultaneously. In contrast, although addition of PAXX super-shifted Ku–DNA bands, it had no effect on Ku–RNA interactions (Figure 4III), suggesting that interaction of Ku with RNA and PAXX is mutually exclusive and RNA and PAXX may bind to similar regions of the Ku70/Ku80 heterodimer. Like RNA, PAXX also promoted Ku dimer formation (Figure 4IV) as observed by SEC-SAXS, although the fraction of the dimer population is smaller as compared to that formed by RNA. The SAXS data for the PAXX dimer alone were of high quality. The atomistic model of full-length PAXX homodimer (see ‘Materials and Methods’ section) matches the SAXS data very well (Figure 4 and Supplementary Figure S13B). The model shows a flexible C-terminus that folds back on itself, which is not observed in the crystal structure (83).

### Ku promotes nuclear retention of LINP1 and LINP1 competes with PAXX *in vivo*

**Figure 5.**
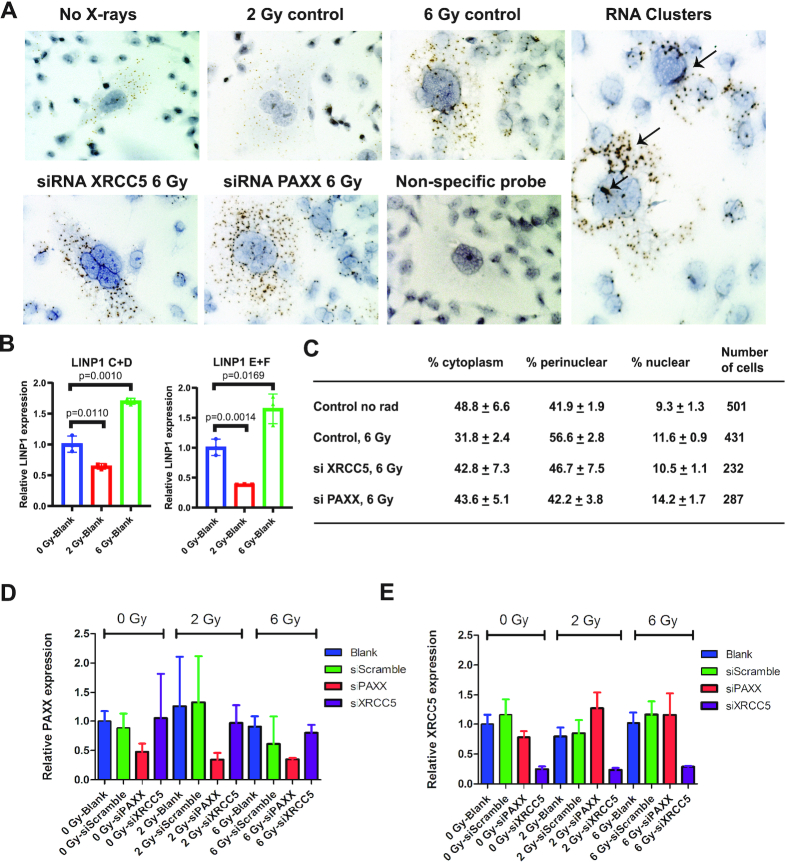
LINP1 localization in MDA-231 cells in response to radiation and XRCC5 and PAXX siRNA knockdowns. (**A**) Single-molecule FISH was performed using RNA-Scope ZZ probes specific for LINP1 lncRNA to monitor changes in LINP1 localization in response to radiation and XRCC5 and PAXX knockdowns. The cells were probed with RNA-Scope ZZ probes 45 min after irradiation. Each foci/dot represents a single molecule of LINP1 and the different intensities are indicative of the different number of probes bound to a single RNA molecule upon hybridization. LINP1 is predominantly cytoplasmic in the absence of X-ray irradiation and becomes more nuclear and perinuclear when cells are irradiated with an X-ray dose of 6 Gy. More cytoplasmic foci and less perinuclear and nuclear foci are observed upon XRCC5 knockdown. Knockdown of PAXX showed a redistribution of LINP1 from the perinuclear space to more nuclear and cytoplasmic staining. No foci were observed when cells were hybridized to a non-specific probe as a control. Large RNA aggregates or clusters are observed (shown by arrows) in most cells in the presence of X-rays indicating that the RNA self associates in cells. (**B**) The change in LINP1 expression in the presence of either 0, 2 or 6 Gy ionizing radiation was quantified by RT-PCR using primer sets C+D and E+F as described in methods. (**C**) LINP1 FISH foci were counted in 230–500 cells for each experiment and the fraction of cytoplasmic, perinuclear and nuclear foci was determined. (**D**) The efficiency of PAXX knockdown was quantified by qPCR in the presence of either 0, 2 or 6 Gy ionizing radiation. A scramble siRNA (see ‘Materials and Methods’ section) was also used as a control. (**E**) The efficiency of XRCC5 (Ku80) knockdown was quantified by qPCR in the presence of either 0, 2 or 6 Gy ionizing radiation. A scramble siRNA (see ‘Materials and Methods’ section) was also used as a control.

To determine if LINP1 and PAXX compete for Ku in cells, we determined LINP1 localization in MDA-231 breast cancer cells using single-molecule RNA fluorescence *in situ* hybridization (FISH) (Figure 5). In the absence of DNA damage, the endogenous LINP1 lncRNA appeared to be a moderately abundant lncRNA (with a *C*_t_ value of ∼22 by qPCR) and was mostly expressed in the cytoplasm and perinuclear space (Figure 5A and C). Significantly higher levels of LINP1 expression were observed in multi-nucleated and in mitotic cells suggesting LINP1 expression maybe related to cell division, proliferation, cell growth and differentiation. We quantified LINP1 levels by RT-PCR (Figure 5B) and found a significant (∼1.8 fold) increase in LINP1 expression when the dose of X-rays was increased from 2 to 6 Gy (Figure 5B). When XRCC5 (Ku80) was knocked down with siRNAs (Figure 5A, C and D), LINP1 was more cytoplasmic and less perinuclear or nuclear, suggesting Ku binding facilitates nuclear recruitment and retention of LINP1. Intriguingly, PAXX knockdown (Figure 5A, C and D) resulted in a redistribution of LINP1 mostly from the perinuclear space to more nuclear and cytoplasmic foci. These data are consistent with LINP1 compensating for PAXX in cells upon induction of double-strand breaks. No foci were observed in the presence of a non-specific FISH probe indicating that the foci are specific for LINP1. Remarkably, we found that large clusters of LINP1 foci formed due to many molecules of LINP1 self-associating to form large aggregates, observed after 45 min in the presence (but not absence) of 6 Gy radiation (Figure 5A). The radiation-induced RNA clusters observed by single-molecule FISH supports our *in vitro* observations of RNA condensates we observed by negative stain EM and SAXS and supports a biological role of RNA–RNA LINP1 interactions during DNA double-strand break repair.

### DNA molecular forceps show LINP1 enhanced stability of the NHEJ synaptic complex

**Figure 6.**
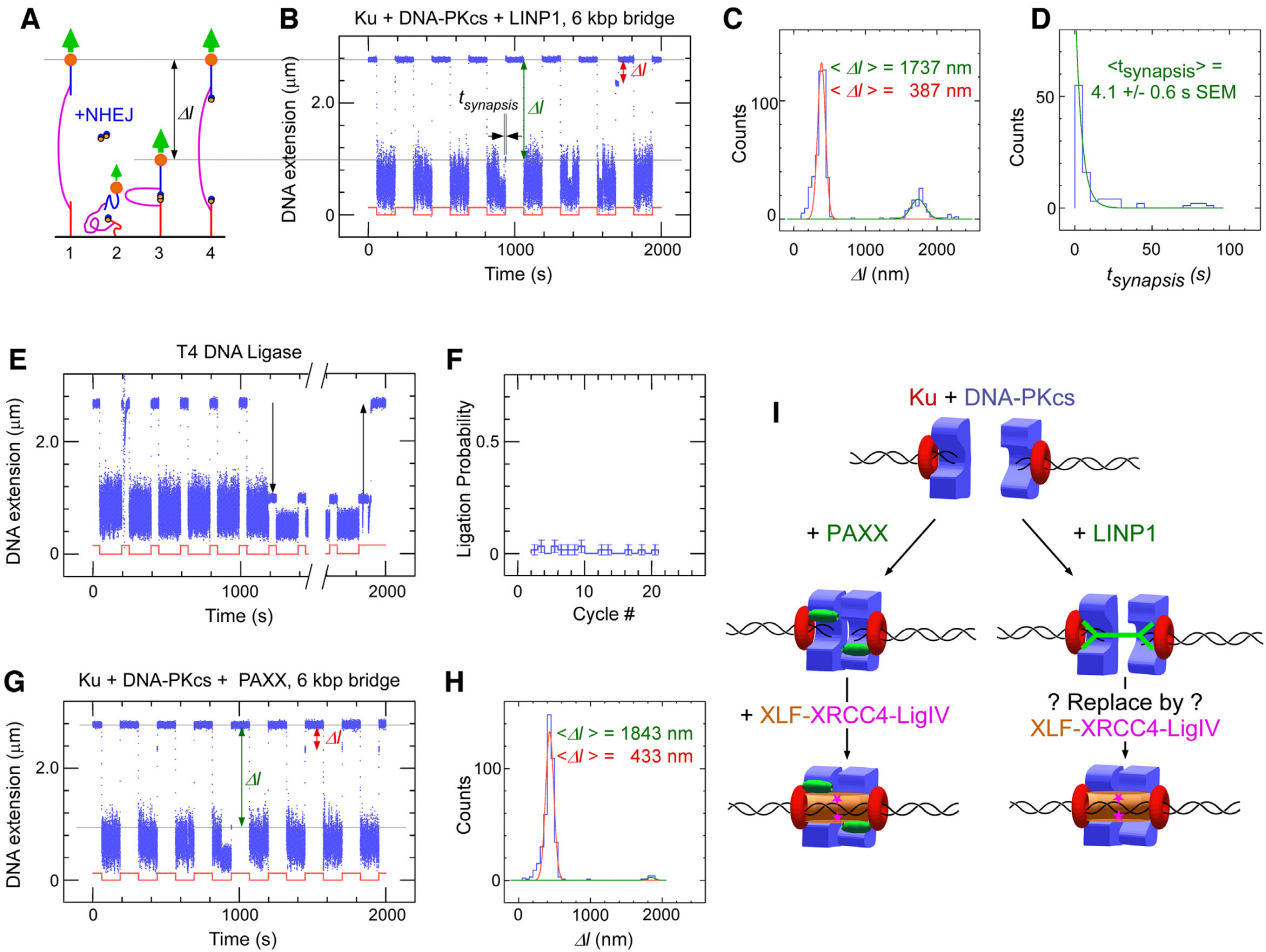
LINP1 is able to promote DNA-PK-dependent synapsis on DNA constructs with a 6 kbp bridge. (**A**) Sketch of the molecular DNA forceps with 6 kbp bridge extended in a magnetic trap. Two ends of the construct are attached to the slide surface and magnetic bead, respectively. The bead is initially held by a 1.4 pN force (1) before the force is lowered to 0.001 pN (2) allowing the two DNA ends to meet in the presence of NHEJ components. The force is then raised again, but any synapsis prevents the bead from recovering its original position (3) until synapsis is broken (4). (**B**) Representative time-trace for Ku + DNA-PKcs + full-length LINP1 obtained upon application of the force-modulation pattern (red). DNA is prepared with blunt ends by SmaI digest. The fourth pulling cycle shows an end-interaction rupture event which can be characterized by both the change in DNA extension upon rupture, *Δl*, and the duration of the synaptic event prior to rupture, *t_synapsis_*. (**C**) Histogram of DNA extension change, *Δl*, upon synapsis rupture in the presence of Ku + DNA-PKcs + full-length LINP1. Green line and red line are from a fit to a double Gaussian distribution, with a peak (green) at 1737 nm and displaying 101 nm standard deviation (n = 95 events), and a peak (red) at 387 nm and displaying 54 nm standard deviation (n = 395 events). The entire histogram contains n = 526 events. (**D**) Lifetime distribution of the specific synaptic state for Ku + DNA-PKcs + full-length LINP1 is fit to a single-exponential distribution (green line), giving a lifetime of 4.1 ± 0.6 s (SEM, n = 95). (**E**) Representative time-trace obtained by force-cycling in the presence of T4 DNA ligase a forceps DNA prepared with sticky ends by XmaI digest. A stable, ∼1.7 μm reduction in extension is observed (down arrow), and is reversed upon introduction of Sma I (up arrow). (**F**) Histogram of ligation events per cycle in the presence of T4 DNA ligase. (**G**) Representative time-trace obtained as in (B) but in the presence of Ku + DNA-PKcs + PAXX. (**H**) Histogram of DNA extension change, *Δl*, upon synapsis rupture in the presence of Ku + DNA-PKcs + PAXX. Green line and red line are from a fit to a double Gaussian distribution, with a peak (green) at 1843 nm displaying a 57 nm standard deviation (n = 10 events), and a peak (red) at 433 nm displaying a 56 nm standard deviation (n = 392 events). (**I**) Schematic model for the role of LINP1 from single-molecule studies.

To determine the functional consequences of the Ku–LINP1 interactions described here, we turned to single-molecule assays based on a novel 11 kbp ‘molecular tweezer’ assembled from a DNA ‘tweezer’ which mimics a DNA double-strand break, as previously described (Figure 6A) (38). In these assays two linear 1.5 kbp dsDNA segments bearing blunt extremities at their tips are positioned face-to-face to mimic the DSB. The segments are held together by a 6 kbp DNA ‘bridge’ covalently anchored to the linear segments 57 bp away from the tips. The linear segments are further tethered, one to an anti-digoxigenin (Dig)-treated glass surface and the other to a streptavidin-coated, 1-μm diameter magnetic bead. Tethering is achieved via 1 kbp biotin- or dig-labeled DNA segments appended to the ends of the 1.5 kbp DNA segments.

The magnetic bead is periodically actuated via a low (1 femtoNewton) or a high (1.4 picoNewton) extending force generated using a magnetic trap, and its position above the surface serves to determine whether physical synapsis is established between DNA ends (Figure 6A). The loss of NHEJ-mediated synaptic interactions between DNA ends is observed as a discrete ‘rip’ in the time-trace representing the bead's position above the glass surface (e.g. Figure 6B), and each ‘rip’ can be characterized by the distribution of its amplitude (Figure 6C) and the time elapsed at high force before it occurs (i.e. the lifetime of the synaptic state, see Figure 6D). Prior experiments have shown that no physical synapsis is established between the DNA ends in the absence of NHEJ components (i.e. no rips are observed), whereas in the presence of DNA-PK only very transient, ∼100 ms synapsis is observed to take place specifically between the DNA ends (38).

In the presence of Ku, DNA-PKcs and LINP1 we readily detect rips with two distinct amplitudes (e.g. red and green arrows, Figure 6B). Accordingly the distribution of rip amplitudes shows two peaks. A major peak is located at ∼400 nm and results from NHEJ-mediated interactions between a DNA tip and the DNA end attached to the proximal surface (38). Because such proximity to the surface can potentially result in additional non-specific interactions, the lifetime of these synaptic events was not considered further. A second, minor peak is located at ∼1700 nm and corresponds to the expected change in DNA extension for a 6 kbp DNA segment extended by a 1.4 pN force (84). The lifetime distribution of synaptic events with this rip amplitude is shown Figure 6D; it is well fit by a single-exponential distribution with a mean lifetime of 4.1 ± 0.6 s (SEM, *n* = 95).

To establish that the 1700 nm peak indeed corresponds to synaptic interactions between the DNA tips, we generated complementary 4-base overhangs at the DNA tips by processing them both with XmaI restriction enzyme. We then applied T4 DNA ligase to the resulting construct (Figure 6E). The observed decrease in DNA extension upon ligation indeed corresponds to ∼1700 nm (down arrow). Ligation was infrequent with this construct, most likely given the long bridge employed here (Figure 6F). Finally, addition of SmaI restriction enzyme re-cleaved the ligated DNA tips and resulted in a 1700 nm increase in DNA extension (up arrow), returning the construct to its initial extension (Figure 6E).

With this same construct, the presence of Ku, DNA-PKcs and PAXX also enables synaptic interactions with two distinct amplitudes as before (Figure 6, panels G and H). In this case, the ∼1700 nm events are comparatively much rarer than when LINP1 was present, suggesting LINP1 is more efficient in bridging distal DNA ends than PAXX. However, the rarity of specific interactions between the DNA tips makes it difficult to analyze their lifetime and compare the stability of synaptic interactions obtained with the two different sets of components.

To quantitatively compare the lifetimes of synapsis made possible by the two different sets of components, we made use of a second DNA construct with a ‘bridge’ length of only 610 bp (Supplementary Figure S15). With this construct synaptic interactions between the DNA tips are frequent with both sets of components, allowing us to collect sufficient data to compare their mean lifetimes (see Table 1). Here synapsis generated by DNA-PK and LINP1 has a mean lifetime of 4.8 ± 0.4 s (SEM, *n* = 357), consistent with the experiments carried out earlier using the 6000 bp bridge construct. On the other hand DNA-PK and PAXX sustains synapsis with a mean lifetime of 2.3 ± 0.1 s (SEM, *n* = 725), consistent with prior measurements (38). We note the lifetime obtained in the presence of DNA-PK, PAXX, XLF and XRCC4 is 63 ± 10 s (SEM, *n* = 146), also as observed in prior measurements (38). Finally, control experiments conducted on the construct with a 610 bp bridge but using Ku, DNA-PKcs, PAXX, XLF, XRCC4 and Ligase IV, and the SL2 or SL2-BT fragments of LINP1 (Supplementary Table S2), show that at 50 μM concentration these fragments inhibit synapsis, with only 0.3% and 1.4% of pulling cycles resulting in synapsis, respectively. This corresponds to a much rarer synapsis than what is observed in the same conditions but without any RNA fragment (22% of pulling cycles), presumably because at the concentrations used the RNAs successfully compete with broken DNA for binding to Ku, as suggested by the measured affinities of Ku for these different components (see Supplementary Table S3).

**Table 1.** Summary of synaptic efficiency, length change (AMP) and lifetime (DUR) at each condition (assay and pulling force)

Assay	Bridge (kbp)	High Force (pN)	Number of pulling cycles	Number of events	Number of Synaptic Events	Efficiency of synapsis	AMP Mean ± SD (nm)	Points	DUR Mean ± SEM (s)
DPK + LINP1	6	1.4	6964	526	95	1.4%	1740 ± 101	95	4.1 ± 0.6
DPK + PAXX	6	1.4	7037	425	10	0.1%	1840 ± 68	10	4.9 ± 4
DPK + LINP1	0.6	1.4	4823	559	357	7.4%	123 ± 20	357	4.8 ± 0.4
DPK + PAXX	0.6	1.4	3562	853	725	20%	141 ± 11	725	2.3 ± 0.1
DPK+PAXX+XLF+LX4	0.6	1.4	1587	350	356	22%	139 ± 10	146	63 ± 10
DPK+PAXX+XLF+LX4+SL2	0.6	1.4	6657	52	22	0.3%	149 ± 19	22	7.6 ± 3.8
DPK+PAXX+XLF+LX4+BT	0.6	1.4	571	12	8	1.4%	159 ± 12	9	1.8 ± 0.6

For each condition, the number of events is determined by counting all pulling cycles in which the DNA extension does not immediately return to the high-extension state when the force is increased. Each event can be characterized by its amplitude (AMP), i.e. the increase in DNA extension observed as the bead recovers its high-extension state at the end of the event, and its duration (DUR), i.e. its observed lifetime. The number of synaptic events corresponds to those events for which a length-change consistent with expectations based on the bridge length and applied force is observed (i.e. for which clear synapsis between DNA tips can be ascertained). Efficiency of synapsis is obtained by dividing the number of synaptic events by the total number of pulling cycles. AMP and DUR for synaptic events are obtained via Gaussian and exponential fitting, respectively (see text for details). The number of events used to obtain the lifetime is specified in the column titled Points; this value can be lower than the number of synaptic events as it does not include events which did not rupture during the pulling cycle in which it was observed, but instead in a successive pulling cycle, making determination of their lifetime difficult to ascertain. We note that the SL2 and BT fragments of LINP1 reduced synaptic efficiency from 22% to 0.3% and 1.4%, respectively, supporting the ability of these fragments to bind NHEJ components. We also note that values cited for conditions yielding < 50 events were obtained by calculating arithmetic averages rather than curve fitting.

## DISCUSSION

The work presented here provides a molecular framework for understanding how chromatin associated lncRNAs may participate in DNA repair processes such as NHEJ, using LINP1-Ku as a model system. We found that Ku is an RNA-binding protein that can interact with structured dsRNAs such as stem-loop structures as well as RNA G-quadruplexes. Previous studies by Jackson and colleagues found that nuclear Ku was released by the addition of RNase (85), suggesting that Ku is bound to RNA in unirradiated cells. Yeast Ku has also been shown to bind to a 48-nt stem loop in TLC1, the RNA component of telomerase, and this interaction is important to recruit and activate telomerase at telomeres (64,65). Given the abundance of Ku heterodimers and of lncRNAs in the nucleus, we suggest that lncRNAs may act as a storage reservoir for Ku until it is recruited to DSBs. Alternatively, Ku may be essential for recruitment of lncRNAs such as LINP1 to double-strand break sites.

In double-strand break repair via classical NHEJ, Ku functions to stabilize the initial synaptic complex. Studies to date have shown that Ku functions as a scaffold in recruitment of repair proteins to DNA, but the role of Ku-RNA interactions in double-strand break repair has not been explored. We find here that Ku interacts with lncRNAs such as LINP1 and that these chromatin-associated lncRNAs likely participate in NHEJ by promoting Ku multimerization across the DNA break, thereby increasing the lifetime of the synaptic complex. We show from biochemical and single-molecule studies *in vitro* and in cells that the Ku–LINP1 interaction effectively replaces the accessory NHEJ protein PAXX (which also causes Ku dimerization) in the NHEJ complex. PAXX forms a ternary complex with Ku and DNA via its association with the Ku70 subunit (82). It remains to be seen whether other lncRNAs act in NHEJ via a similar mechanism and if competition for Ku by lncRNAs and PAXX has other biological implications.

The importance of structure (or lack of) in lncRNA function remains an enigma, and it is a difficult question to answer due to their large size, flexible nature and non-catalytic activities. Although SHAPE and chemical probing techniques have mapped several lncRNA secondary structures (86–88), information about their 3D architectures is currently lacking. The crystal structure of the 3′ end of MALAT1 reveals a triple helix with the 3′ end sequestered in a U-A-U base triple, protecting the 3′ end from nucleolytic degradation (89). However, there are no structures of intact lncRNAs. Recent SAXS studies of the lncRNA *Braveheart* (90) reveal that, like LINP1 it is flexible in solution; however, it has a well-defined structure that undergoes a conformational change upon binding the zinc finger protein CNBP. This is unlike LINP1, which has secondary structure and is flexible, but is prone to RNA–RNA interactions *in vitro* as well as in cells.

We found that RNA–RNA interactions mediated by flexible lncRNAs can drive the formation of phase-separated condensates. The observation of LINP1 condensates by negative stain EM supports and extends RNA aggregation observed in SAXS experiments for FL LINP1 and LINP1 (1–300), as well as single-molecule RNA-FISH experiments showing an increase in LINP1 abundance upon irradiation. These condensates or aggregates were too small to be observed by light microscopy with Cy3-labeled LINP1. However they appear suitable to represent nucleating components of larger phase-separated foci that are important for efficient DSB break repair by sequestering DNA repair proteins. Trans RNA–RNA interactions are known to be important for driving self-assembly of stress granules (91,92) and specific regions of lncRNAs such as NEAT1 drive paraspeckle formation (93). RNA secondary structure is a major driver of the composition and identity of phase-separated granules (94,95). In neurological diseases such as ALS/FTD, a rG4C2 repeat sequence that likely forms a G-quadruplex is essential for promoting phase-separated granule assembly (96). Future studies will test the role of Ku–RNA and specifically Ku–G-quadruplex interactions in regulating phase-separated DNA repair foci at double-strand break sites *in vivo*. Here, we unveil LINP1 solution structure and phase-separation promoting assemblies along with its Ku–LINP1 interaction and activity in replacing the protein PAAX to stabilize the initial synaptic event for efficient NHEJ.

## Supplementary Material

gkaa784_Supplemental_File
