# Mechanism of efficient double-strand break repair by a long non-coding RNA

**DOI:** 10.1093/nar/gkaa1233

**Published:** 2020-12-18

**Authors:** Roopa Thapar, Jing L Wang, Michal Hammel, Ruiqiong Ye, Ke Liang, Chengcao Sun, Ales Hnizda, Shikang Liang, Su S Maw, Linda Lee, Heather Villarreal, Isaac Forrester, Shujuan Fang, Miaw-Sheue Tsai, Tom L Blundell, Anthony J Davis, Chunru Lin, Susan P Lees-Miller, Terence R Strick, John A Tainer

**Affiliations:** Department of Molecular and Cellular Oncology, University of Texas M.D. Anderson Cancer Center, Houston, TX 77030, USA; Ecole Normale Supérieure, IBENS, CNRS, INSERM, PSL Research University, Paris 75005, France; Molecular Biophysics and Integrated Bioimaging, Lawrence Berkeley National Laboratory, 1 Cyclotron Rd, Berkeley, CA 94720, USA; Department of Biochemistry and Molecular Biology, Robson DNA Science Centre, Charbonneau Cancer Institute, University of Calgary, Alberta, T2N 4N1, Canada; Department of Molecular and Cellular Oncology, University of Texas M.D. Anderson Cancer Center, Houston, TX 77030, USA; Department of Molecular and Cellular Oncology, University of Texas M.D. Anderson Cancer Center, Houston, TX 77030, USA; Department of Biochemistry, University of Cambridge, 80 Tennis Court Road, Cambridge CB2 1GA, UK; Department of Biochemistry, University of Cambridge, 80 Tennis Court Road, Cambridge CB2 1GA, UK; Biological Systems and Bioengineering, Lawrence Berkeley National Laboratory, Berkeley, CA, 94720, USA; Department of Biochemistry and Molecular Biology, Robson DNA Science Centre, Charbonneau Cancer Institute, University of Calgary, Alberta, T2N 4N1, Canada; CryoEM Core at Baylor College of Medicine, Houston, Texas 77030, USA; CryoEM Core at Baylor College of Medicine, Houston, Texas 77030, USA; Department of Biochemistry and Molecular Biology, Robson DNA Science Centre, Charbonneau Cancer Institute, University of Calgary, Alberta, T2N 4N1, Canada; Biological Systems and Bioengineering, Lawrence Berkeley National Laboratory, Berkeley, CA, 94720, USA; Department of Biochemistry, University of Cambridge, 80 Tennis Court Road, Cambridge CB2 1GA, UK; Division of Molecular Radiation Biology, Department of Radiation Oncology, UT Southwestern Medical Center, Dallas, TX 75390, USA; Department of Molecular and Cellular Oncology, University of Texas M.D. Anderson Cancer Center, Houston, TX 77030, USA; Department of Biochemistry and Molecular Biology, Robson DNA Science Centre, Charbonneau Cancer Institute, University of Calgary, Alberta, T2N 4N1, Canada; Ecole Normale Supérieure, IBENS, CNRS, INSERM, PSL Research University, Paris 75005, France; Programme “Equipe Labellisée’’, Ligue Nationale Contre le Cancer, Paris 75005, France; Department of Molecular and Cellular Oncology, University of Texas M.D. Anderson Cancer Center, Houston, TX 77030, USA; Molecular Biophysics and Integrated Bioimaging, Lawrence Berkeley National Laboratory, 1 Cyclotron Rd, Berkeley, CA 94720, USA; Department of Cancer Biology, University of Texas M.D. Anderson Cancer Center, Houston, TX 77030, USA

*Nucleic Acids Research*, Volume 48, Issue 19, 4 November 2020, Pages 10953–10972, https://doi.org/10.1093/nar/gkaa784

The Authors wish to correct an error in Figure 6. In Figure 6B the panel label should read ‘Ku + DNA-PKcs + **LINP1**, 6 kbp bridge’ instead of ‘Ku + DNA-PKcs + **PAXX**, 6 kbp bridge’.

**Figure 6.**
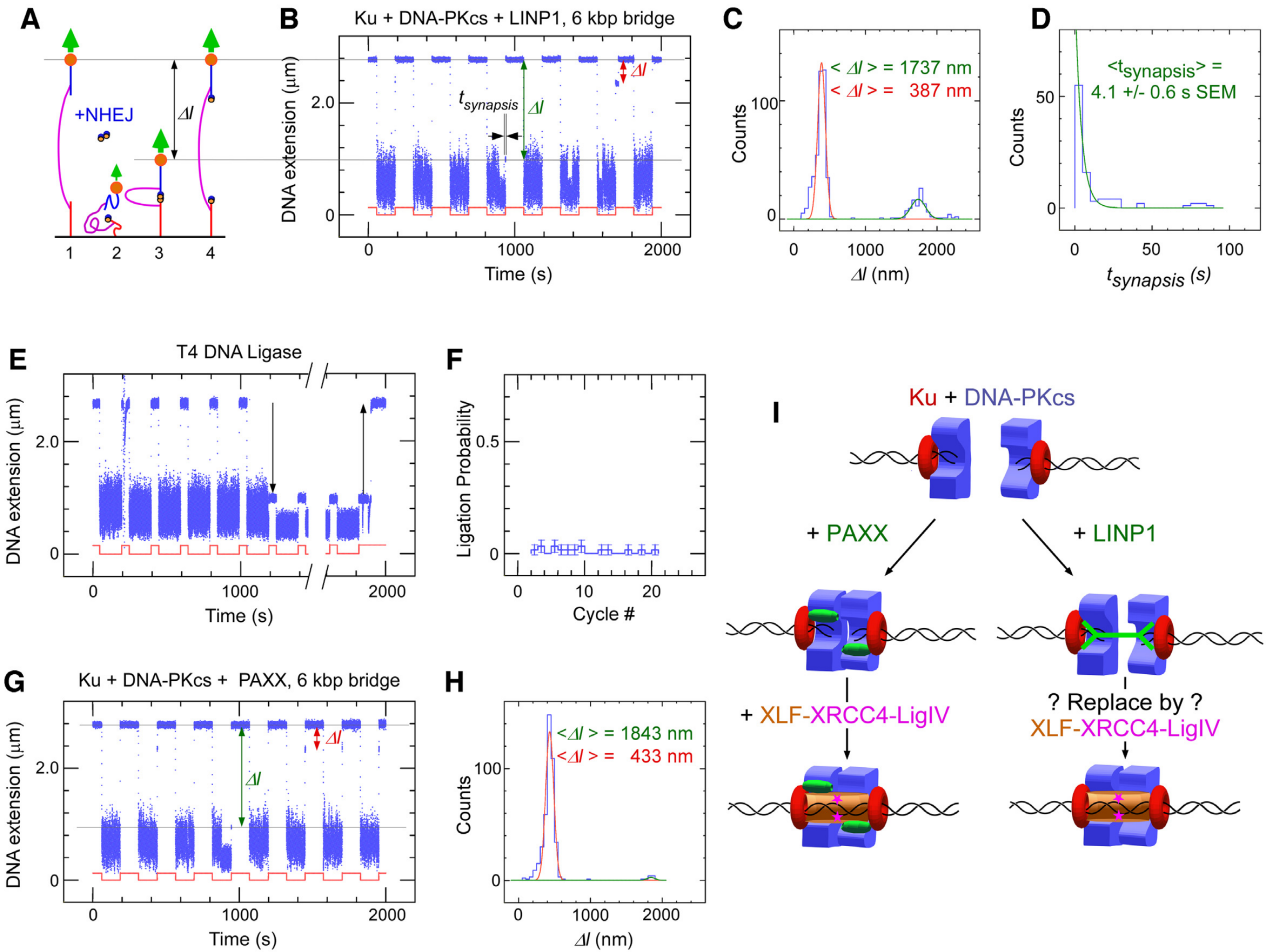
LINP1 is able to promote DNA-PK-dependent synapsis on DNA constructs with a 6 kbp bridge. (**A**) Sketch of the molecular DNA forceps with 6 kbp bridge extended in a magnetic trap. Two ends of the construct are attached to the slide surface and magnetic bead, respectively. The bead is initially held by a 1.4 pN force (1) before the force is lowered to 0.001 pN (2) allowing the two DNA ends to meet in the presence of NHEJ components. The force is then raised again, but any synapsis prevents the bead from recovering its original position (3) until synapsis is broken (4). (**B**) Representative time-trace for Ku + DNA-PKcs + full-length LINP1 obtained upon application of the force-modulation pattern (red). DNA is prepared with blunt ends by SmaI digest. The fourth pulling cycle shows an end-interaction rupture event which can be characterized by both the change in DNA extension upon rupture, *Δl*, and the duration of the synaptic event prior to rupture, *t_synapsis_*. (**C**) Histogram of DNA extension change, *Δl*, upon synapsis rupture in the presence of Ku + DNA-PKcs + full-length LINP1. Green line and red line are from a fit to a double Gaussian distribution, with a peak (green) at 1737 nm and displaying 101 nm standard deviation (n = 95 events), and a peak (red) at 387 nm and displaying 54 nm standard deviation (n = 395 events). The entire histogram contains n = 526 events. (**D**) Lifetime distribution of the specific synaptic state for Ku + DNA-PKcs + full-length LINP1 is fit to a single-exponential distribution (green line), giving a lifetime of 4.1 ± 0.6 s (SEM, n = 95). (**E**) Representative time-trace obtained by force-cycling in the presence of T4 DNA ligase a forceps DNA prepared with sticky ends by XmaI digest. A stable, ∼1.7 μm reduction in extension is observed (down arrow), and is reversed upon introduction of Sma I (up arrow). (**F**) Histogram of ligation events per cycle in the presence of T4 DNA ligase. (**G**) Representative time-trace obtained as in (B) but in the presence of Ku + DNA-PKcs + PAXX. (**H**) Histogram of DNA extension change, *Δl*, upon synapsis rupture in the presence of Ku + DNA-PKcs + PAXX. Green line and red line are from a fit to a double Gaussian distribution, with a peak (green) at 1843 nm displaying a 57 nm standard deviation (n = 10 events), and a peak (red) at 433 nm displaying a 56 nm standard deviation (n = 392 events). (**I**) Schematic model for the role of LINP1 from single-molecule studies.

The published article has been updated. This error does not affect the conclusions of the article.

