# Supplementary material for: Mechanism of efficient double-strand break repair by a long non-coding RNA
Source: Nucleic Acids Res. 2020 Oct 12;48(19):10953–72. doi: 10.1093/nar/gkaa784 (PMC7641761; doi:10.1093/nar/gkaa784)
Supplement: gkaa784_Supplemental_File [file gkaa784_supplemental_file.pdf]

KEY RESOURCES TABLE

REAGENT or RESOURCE	SOURCE	IDENTIFIER
**Biological samples**		
**Chemicals, Peptides and Recombinant Proteins**		
HeLa cell Fractions	Abcam	Ab168542
RNaseOUT	Invitrogen	# 10777019
Hela Nuclear Extracts	IPRATECH	
DNA-PKcs	Purified from 100L HeLa cells	PMID:15177184
Ku70/ His-Ku80	Purified from baculovirus-infected insect cells	PMID:28641126
PAXX	Expressed in E coli as GST fusion. GST removed with PreScission Protease	
**Antibodies**		
Rabbit polyclonal to gamma H2A.X (phospho S139)	Abcam	Ab11174
Rabbit Polyclonal anti-PAXX	Abcam	Ab216353
Mouse Monoclonal anti-KU80	Abcam	Ab119935
DNA-PKcs	Rabbit polyclonal	SPLM in house
Ku80	Abcam	Ab3107
XLF	Rabbit polyclonal	**Described in** PMID: 18644470
XLF pS245	Affinity purified	**Described in** PMID: 18644470
**Commercial Assays and Reagents**		
Superdex 200 HiLoad 16/300 GL	GE Healthcare	17-5175-01

HisTrap HP 5 mL	GE Healthcare	17-5248-02
HiTrap Q HP 5 mL	GE Healthcare	17115401
Shodex KW803	Shodex	F6989103
HiLoad Superdex 200 16/600	GE Healthcare	28989335
Oligonucleotides	Table S2	N/A
Recombinant DNA		
LINP1-pCMV 3Tag-3a pcDNA3.1/Hygro(+)	Genescript	U7764EE140-3
LINP1-pCMV 3Tag-3a pCMV-3Tag-3a	Genescript	U7764EE140-2
pEGFP-N1	Clonetech	Discontinued
**Software and algorithms**		
ScÅtter	https://bl1231.als.lbl.gov/scatter/	N/A
Astra 7.1	https://www.wyatt.com/products/software/astra.html	N/A
Pymol	https://pymol.org/2/	N/A
Chimera	https://www.cgl.ucsf.edu/chimera	N/A
Image lab for Chemidoc^{TM} MP	https://www.bio-rad.com/en-us/product/image-lab-software?ID=KRE6P5E8Z	N/A
BILBOMD	https://bl1231.als.lbl.gov/bilbomd	N/A
FOXS	https://modbase.compbio.ucsf.edu/fox	N/A

	s/	
MultiFOXS	http://modbase.compbio.ucsf.edu/multifoxs/	N/A
Picotwist	http://www.picotwist.com/	N/A

Oligos qPCR

LINP1 a	GCTCGCATATCTCCACTTGC	IDT
LINP1 b	GCTCTGTTCTGGGTGACACT	IDT
LINP1 c	AGCCGGTCCAGTACACCTTT	IDT
LINP1 d	GGAAAGCACCGTCTGTTGTT	IDT
LINP1 e	CCCGAAATTCAAGCCACACA	IDT
LINP1 f	TCCCCATACCCTCTCCTACC	IDT
DBCO-Hyb1	TCCATGGGCATACTGATCGGTAGGG	TriLink
DBCO-Hyb2	TGAGCCAAGACGCCTCCATCCATGCA	TriLink
Az-Charomid-1456-SmaI	GAGAGACCCGGGCACCGTCTCCTTCGAACTTATTCGCAATGGAGTGTCATTCATCAAGGACGCCGC (AmC6-T/Azido) ATCGCAAATGGTGCTATCC	TriLink
Az-Charomid-3778-SmaI	GAGAGACCCGGGCACGACTTATCGCCACTGGCAGCAGCCACTGGTAAAGGATTAGCAGAGCGAGG (AmC6-T/Azido) ATGTAGGCGGTGCTACAGAG	TriLink
O1-Comp	pho-CGCGCCCTACCGATCAGTATGCCCATGGA	TriLink
O2-Comp	pho-TGGATGGAGGCGTCTTGGCTCA	TriLink

ON-TARGETPLUS SMARTPOOL siRNAs

PAXX	CAGGAGAGUCGCUCAUCAA<br>UAACACGGCUCCUCAAAUU<br>CGGGUUCAAGAGUAAGAAA<br>CAGCUCCCUUGGUUGGAAA	Dharmacon<br>L-026038-02-0005
XRCC5	GCAUGGAUGUGAUUCAACA<br>GAGCAGCGCUUUAACAACU<br>CGAGUAACCAGCUCAUAAA<br>AAACUUCCGUGUUCUAGUG	Dharmacon<br>L-010491-00-0005

Non-targeting control	UGGUUUACAUGUCGACUAA<br>UGGUUUACAUGUUGUGUGA<br>UGGUUUACAUGUUUUCUGA<br>UGGUUUACAUGUUUUCCUA	Dharmacon<br>D-001810-10-05

LEAD CONTACT

Further information and requests for resources should be directed to and will be fulfilled by the Lead Contact, John A. Tainer (jatainer@gmail.com).

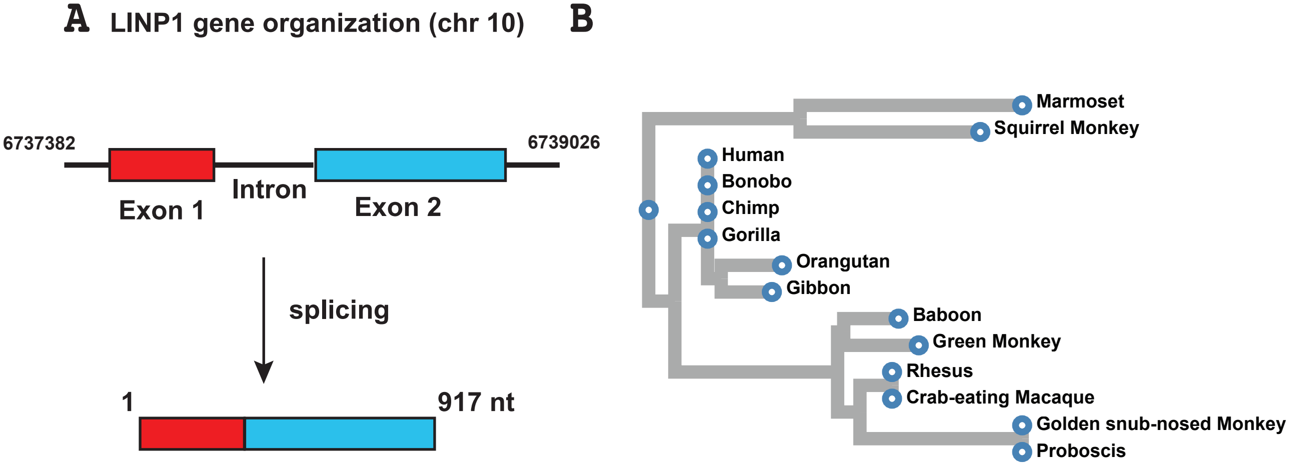

Figure S1. Domain organization and phylogenetic conservation of LINP1. (A) LINP1 spans a region from nts 6737382 - 6739026 on chr 10 in the human genome. The 917 nt mature ncRNA is transcribed from two exons and the intron between 6737629 - 6738356 nts is spliced out. (B) LINP1 is conserved only in primates. A simplified phylogenetic tree showing LINP1 orthologues in primates is shown.

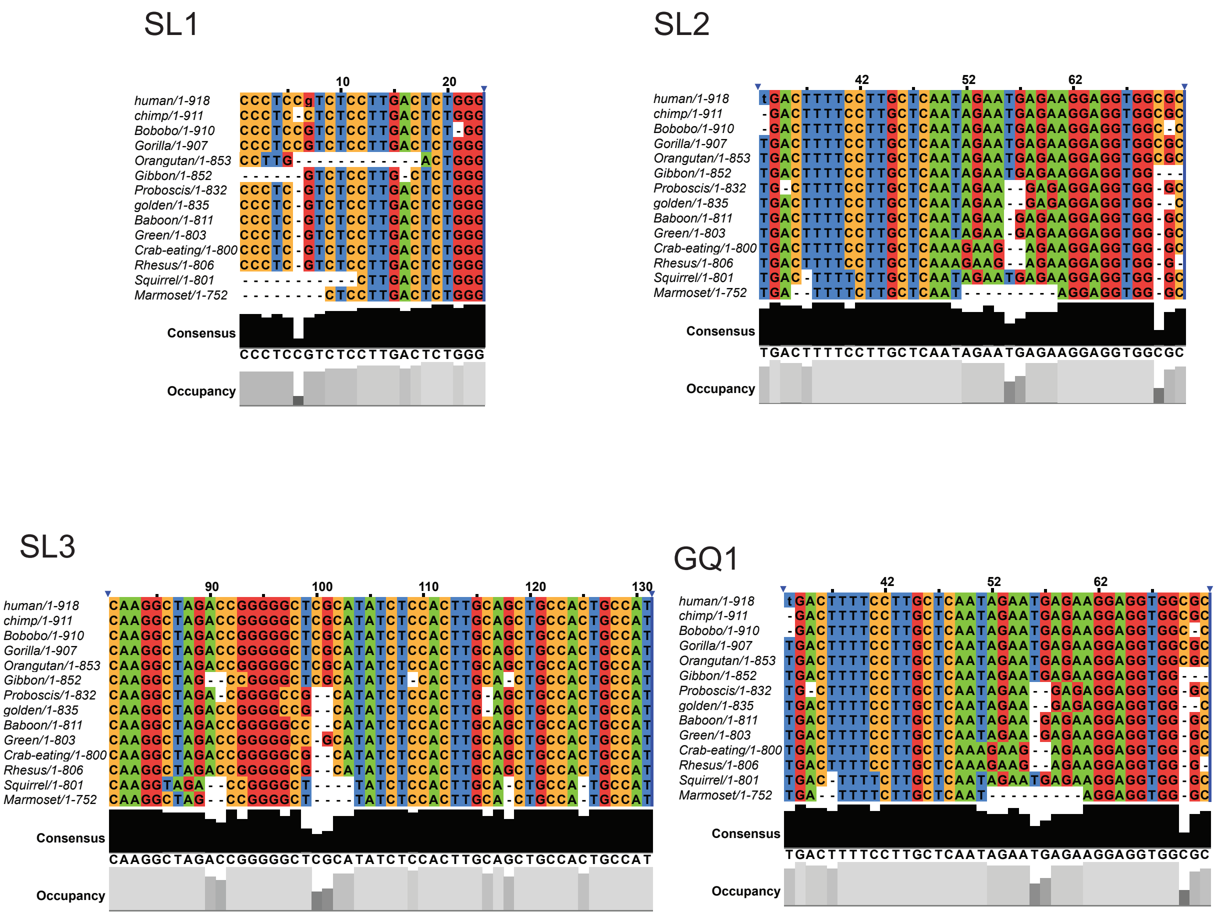

Figure S2. Sequence conservation of stem-loops SL1 (human sequence nts 1-23), SL2 (human sequence nts 33-72), SL3 (human sequence nts 81-130) and the G-quadruplex (human sequence nts 611-645) GQ1 across primates. The alignment of the sequences is presented and color-coded by nucleotide type. The consensus is shown at the bottom.

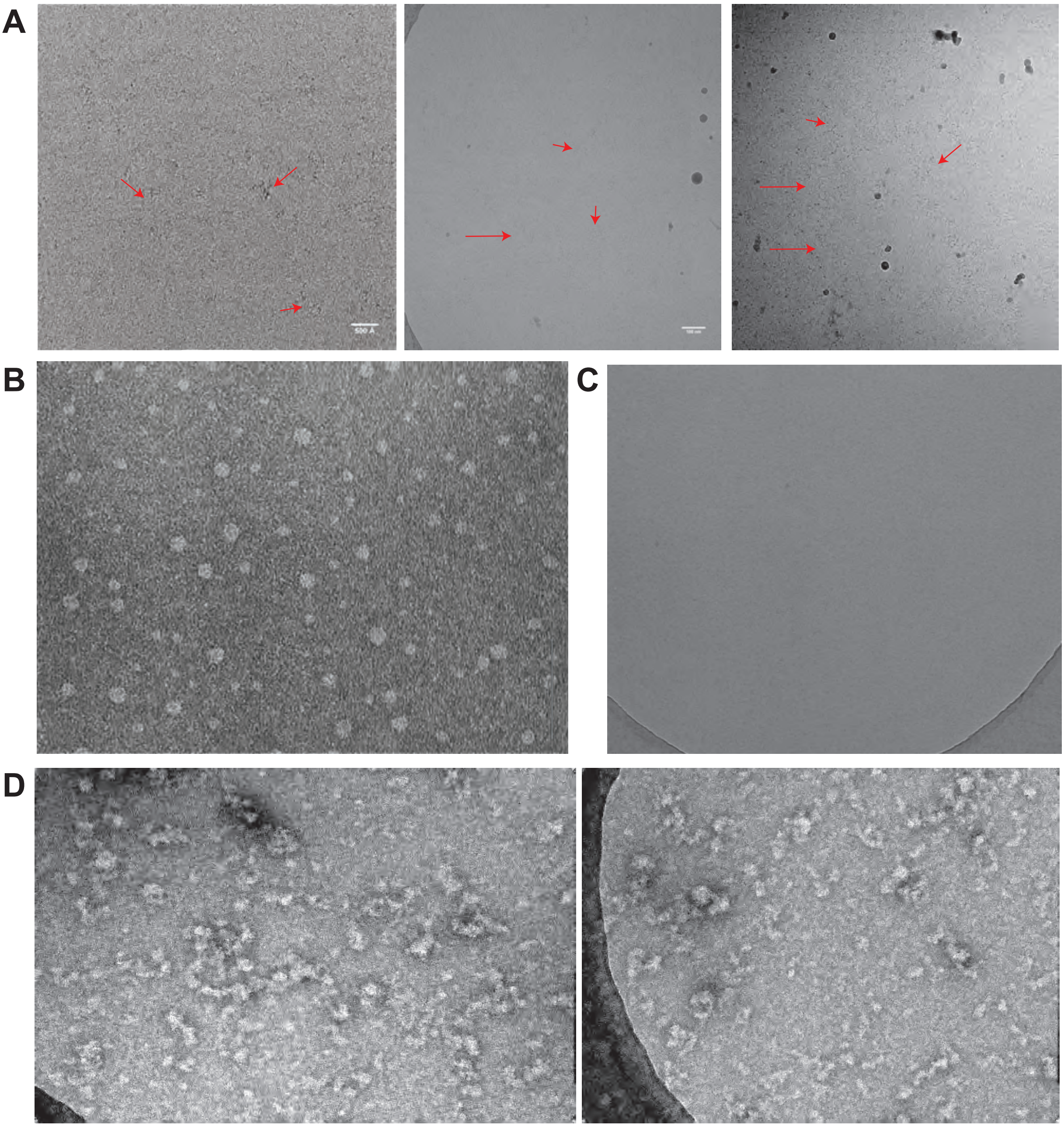
A
B
C
D
500 Å

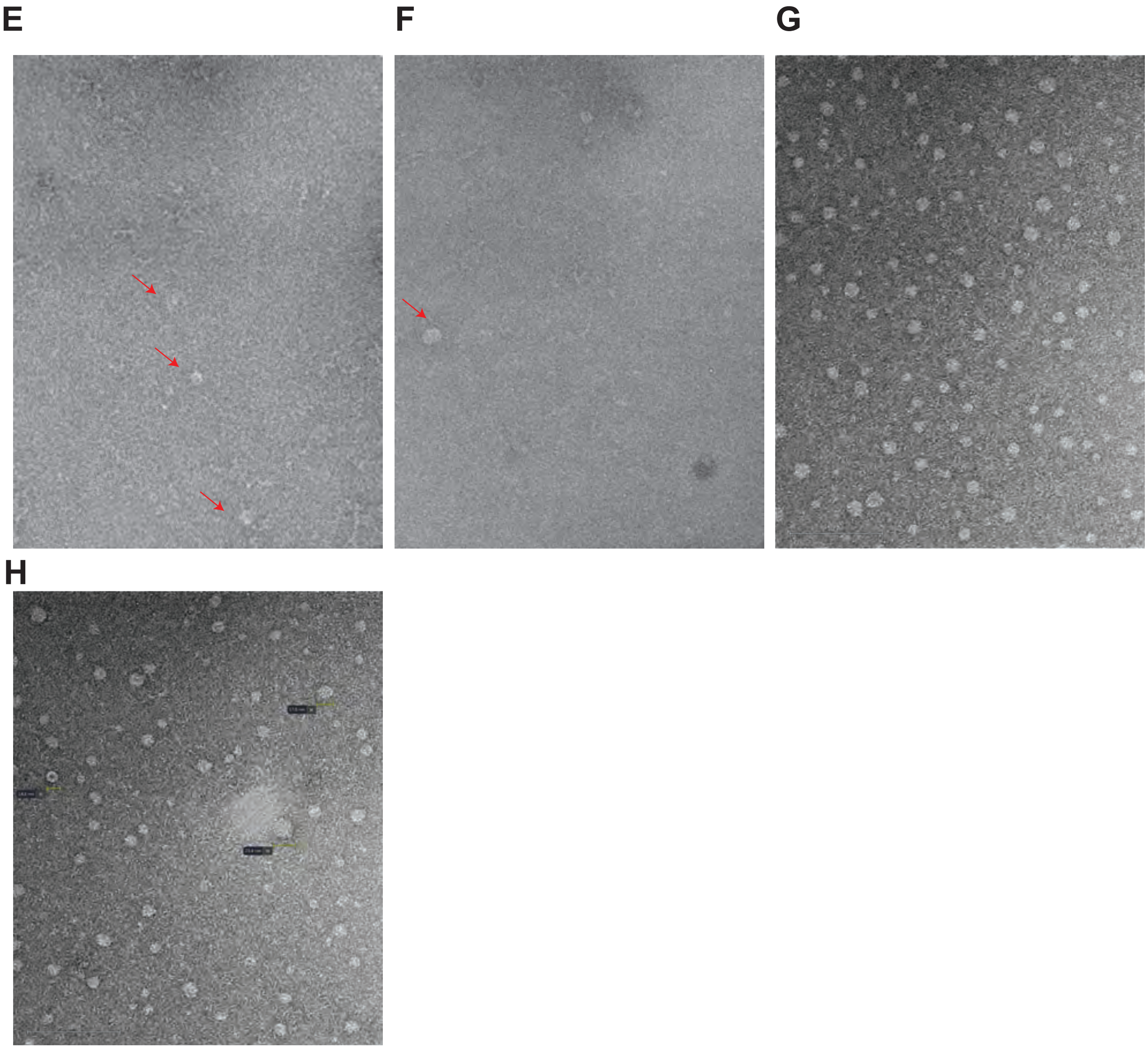

Figure S3. Cyro-EM and Negative Stain EM images of FL LINP1 and LINP1 (1-300)
(A) Cryo-electron microscopy (EM) of FL LINP1 at 0.05, 0.1, and 0.2 mg/ml. Images in three panels show flexible LINP1 particles that are not electron dense in three different experiments. (B) Negative stain EM of FL LINP1 at 0.1 mg/ml in the presence of 0.5% PEG400 show round electron-dense droplets between 10 - 24 nm in size. (C) No droplets were observed in the buffer control. (D) Addition of FL Ku to FL LINP1 shows oligomerization and filamentous Ku-LINP1 complex structures. (E, F) Negative stain EM images of LINP1 (1-300) at 0.05 and 0.1 mg/ml showing a equilibrium between RNA droplets (red arrows) and flexible LINP1particles. (G, H) At concentrations between 0.2 mg/ml - 0.5 mg/ml, the LINP1 (1-300) forms RNA droplets in the absence of PEG400.

A

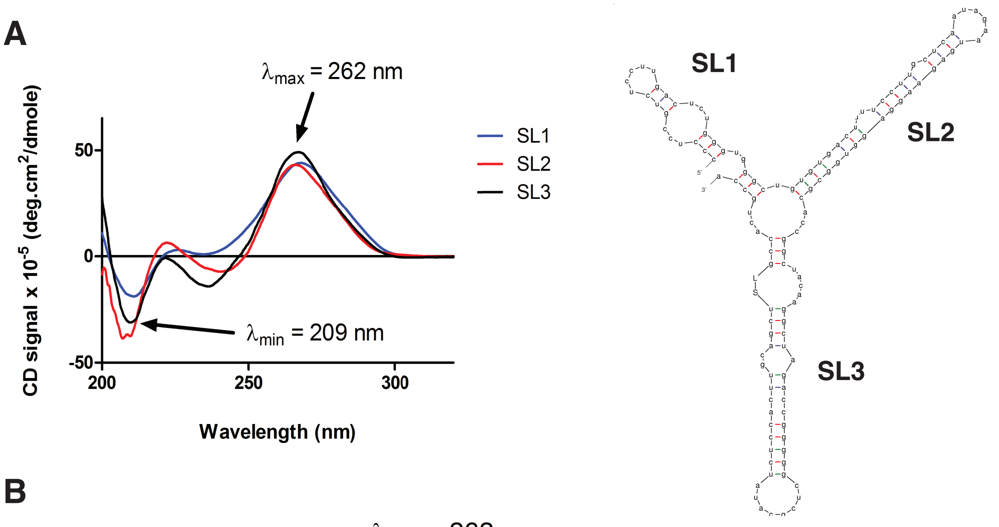

B

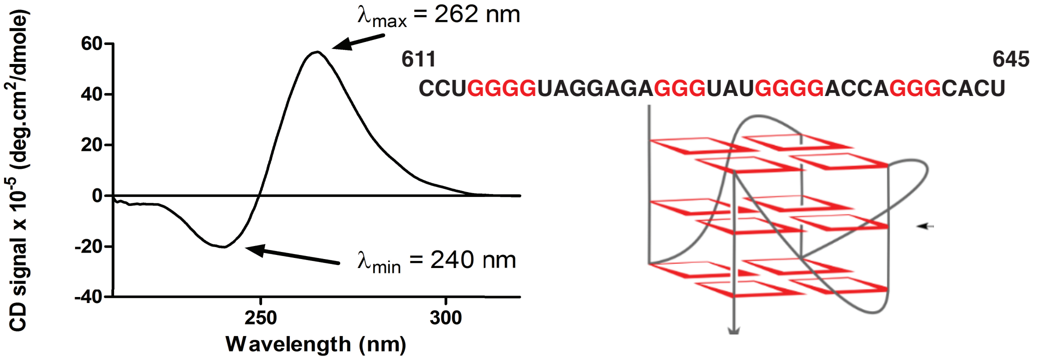

C

Thermal melting transitions for LINP1 RNA hairpins and GQ motif

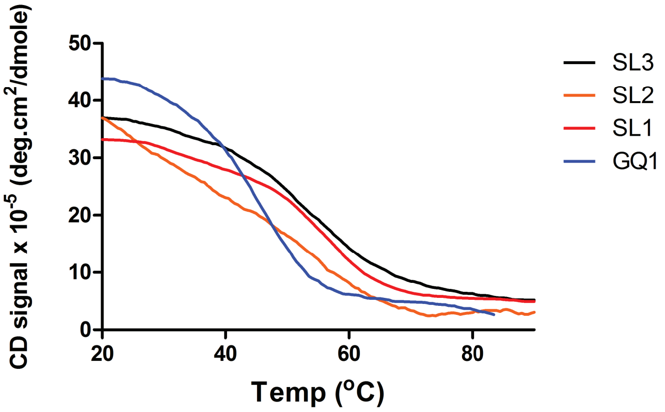

Figure S4.

Circular dichroism (CD) spectra of the stem-loops have a λmax and λmin expected for a hairpin structure and the thermal melting transitions indicates that they adopt a stable fold in solution. CD spectra of the GQ1 motif of LINP1 RNA in the presence of potassium is characteriztic of a parralel G-quadruplex.

S5. Comparison of 1D imino 1H NMR spectra of LINP1 domains at 600 MHz and pH 7.0

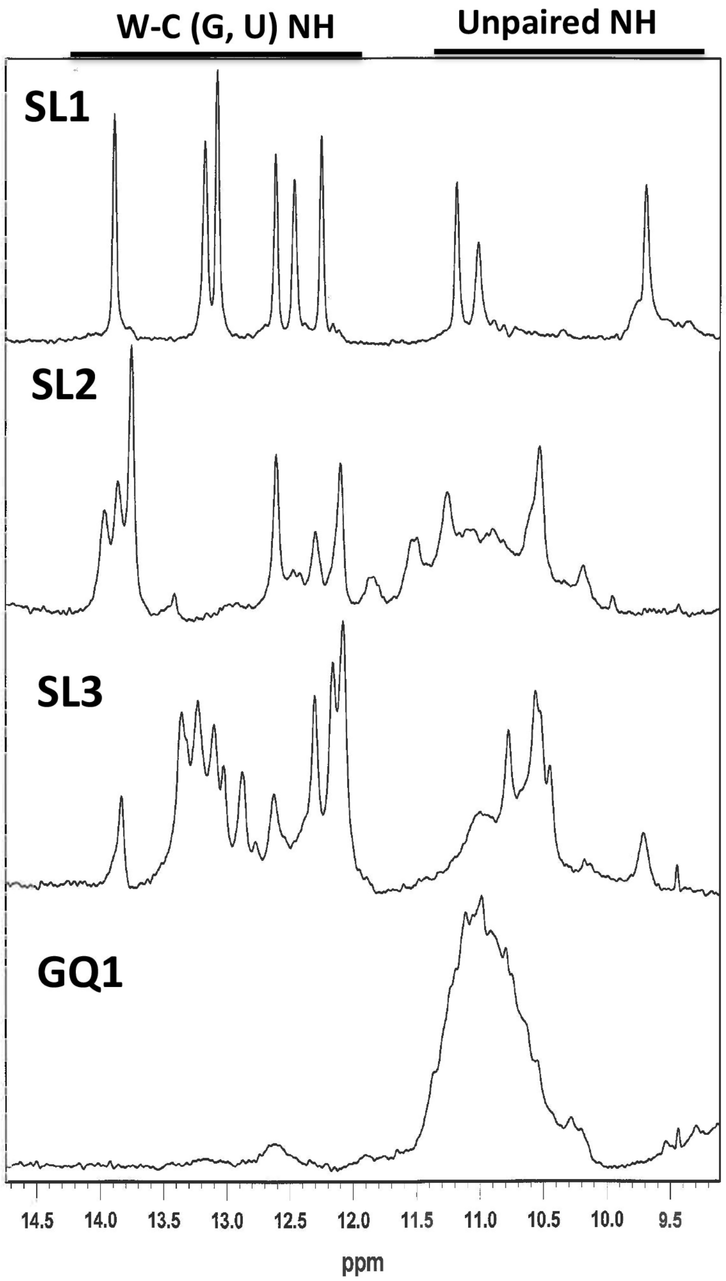

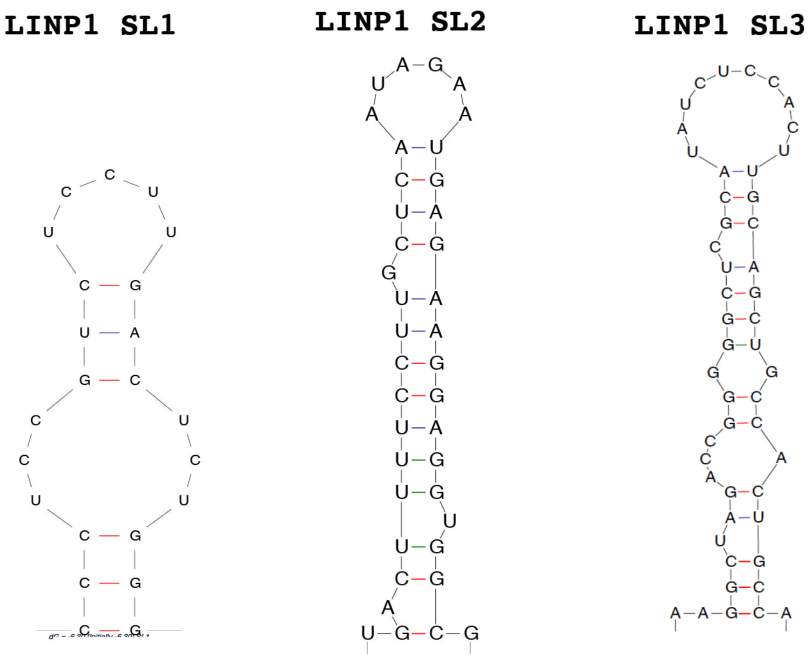

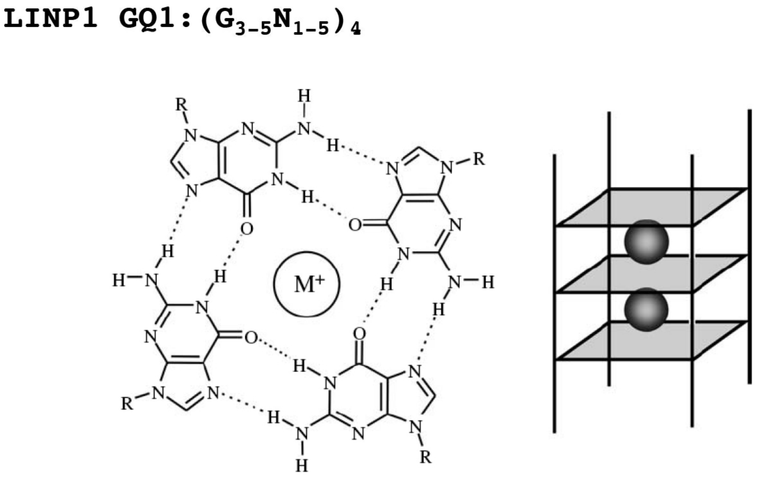

Figure S5. The 1D imino proton NMR spectrum (Figure S6) of the SL1 stem−loop RNA shows six strong resonances in the W−C base-paired region (12−15 ppm) corresponding to the stem of the hairpin as well as three unpaired NH resonances between 9.5-11 p.p.m that correspond to unpaired uridines from the loop and bulge regions of SL1. Similarly, the imino NMR spectra of SL2 RNA shows broader line-widths compared to SL1 (due to increased molecular size) and at least 13/17 imino protons for U and G resonances are observed with strong intensity in the W-C region.
Eight additional resonances are observed in the unpaired region (9.5-11 p.p.m), that likely reflect uridine iminos from the loop, bulge and non W-C base pairs in the stem. The 1D imino NMR spectrum of SL3 shows 12/15 U, G iminos that are W-C paired and at least 10 resonances for unpaired NHs.

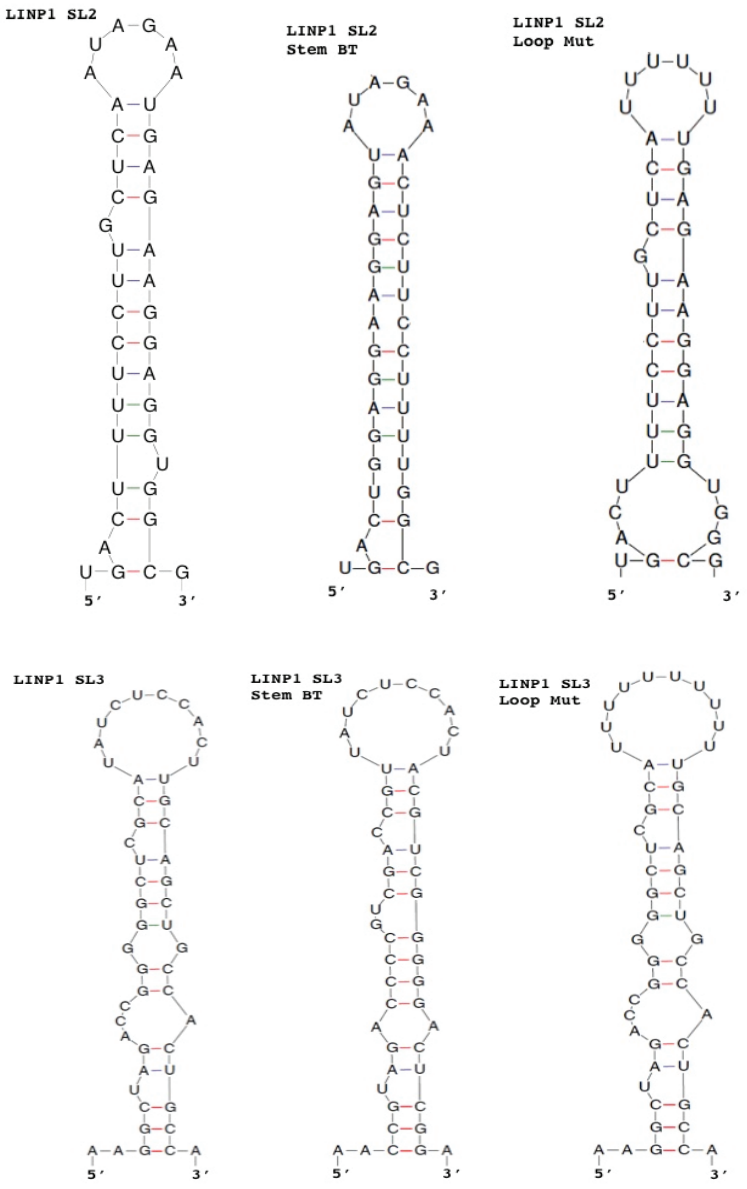

Figure S6. Mfold predicted structures for the SL2 and SL3 loop (LM) and the loop base transversion (BT) mutants .

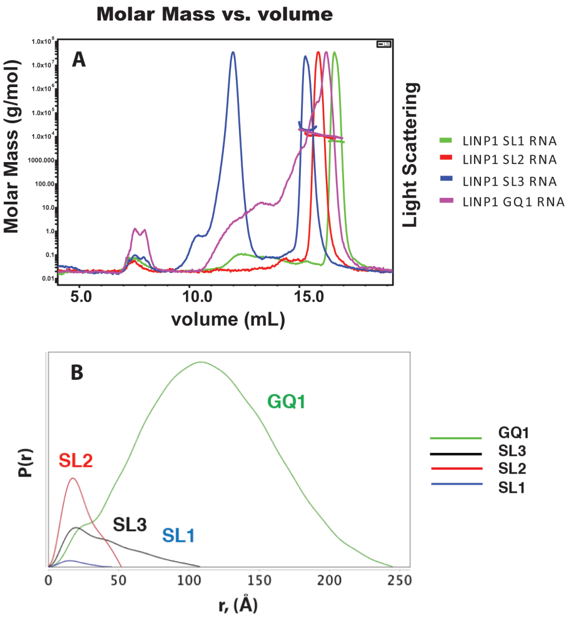

Figure S7

Molecular characterization of LINP1 domains by SEC-MALS and SAXS.
(A) Size exclusion chromatogram from multi-angle light scattering SEC-MALS data for the G-quadruplex (GQ1) and RNA stem-loops SL1, SL2, and SL3 are shown. The GQ1 RNA exists as a polydisperse species (magenta curve) that elutes in a broad range of molecular weights.

(B) Normalized P(r) plots calculated from the experimental SAXS data for the G-quadruplex (GQ1) and RNA stem-loops SL1, SL2, and SL3 are shown. The data shows the large Dmax for the GQ1 RNA indicative of aggregation due to RNA-RNA interactions.

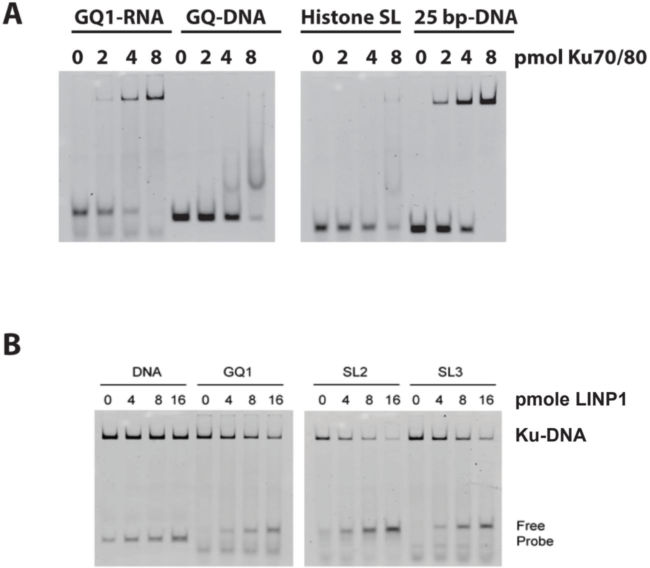

Figure S8. (A) EMSA experiments towards RNA and DNA substrates showing that Ku is specific for RNA G-quadruplexes and has lower affinity for the histone H3 stem-loop that has a short six bp stem and lacks a bulge. (B) RNA is inefficient in competing a pre-formed Ku-DNA complex, likely due to the 10 fold higher affinity of Ku for DNA vs. RNA.

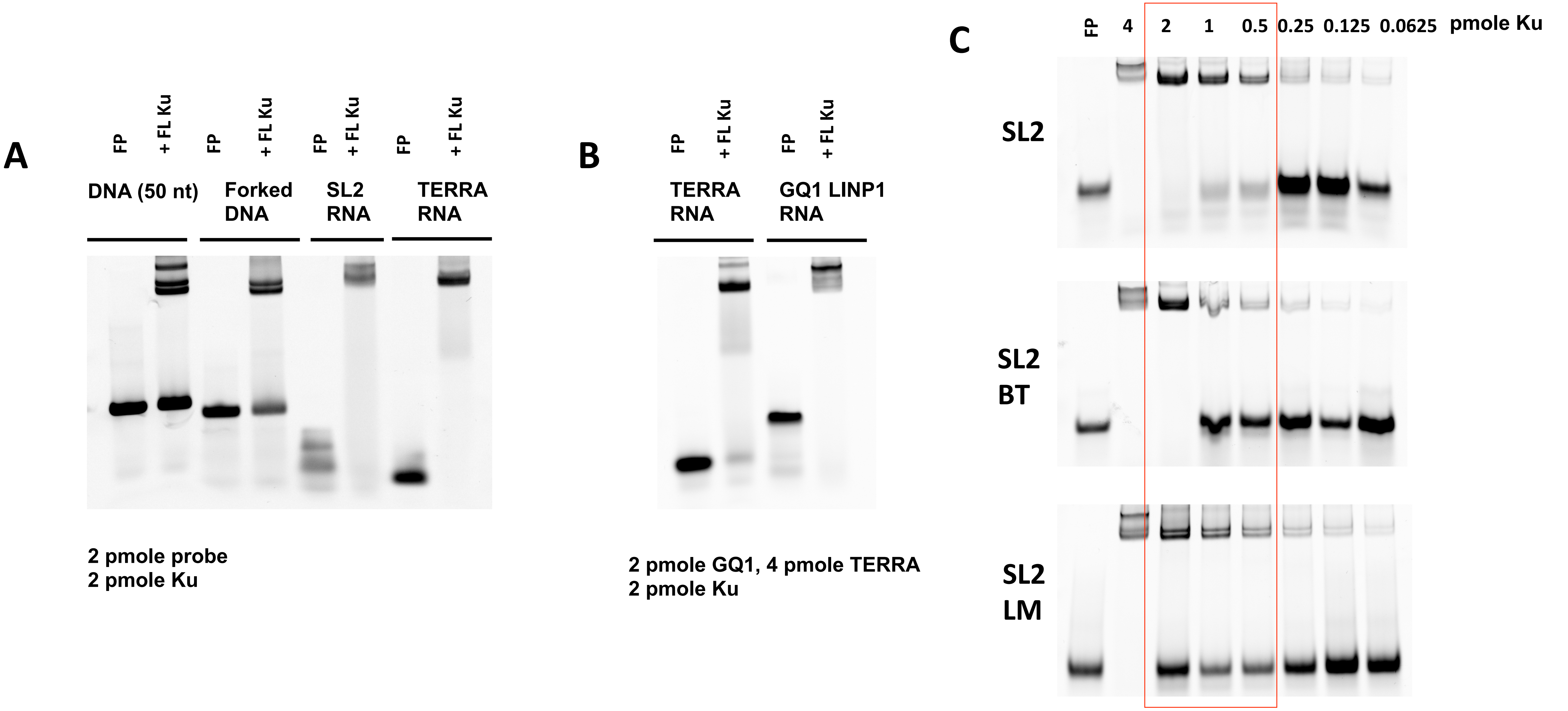

Figure S9. (A and B) EMSA assays to compare binding of Ku to TERRA and LINP1 GQ1 G-quadruplexes to DNA and the LINP1 SL2 RNAs. **(C)** Comparison of Ku binding to SL2, SL2-BT, and SL2 LM by EMSA shows weaker binding of the mutants to Ku compared to the SL2 RNA.

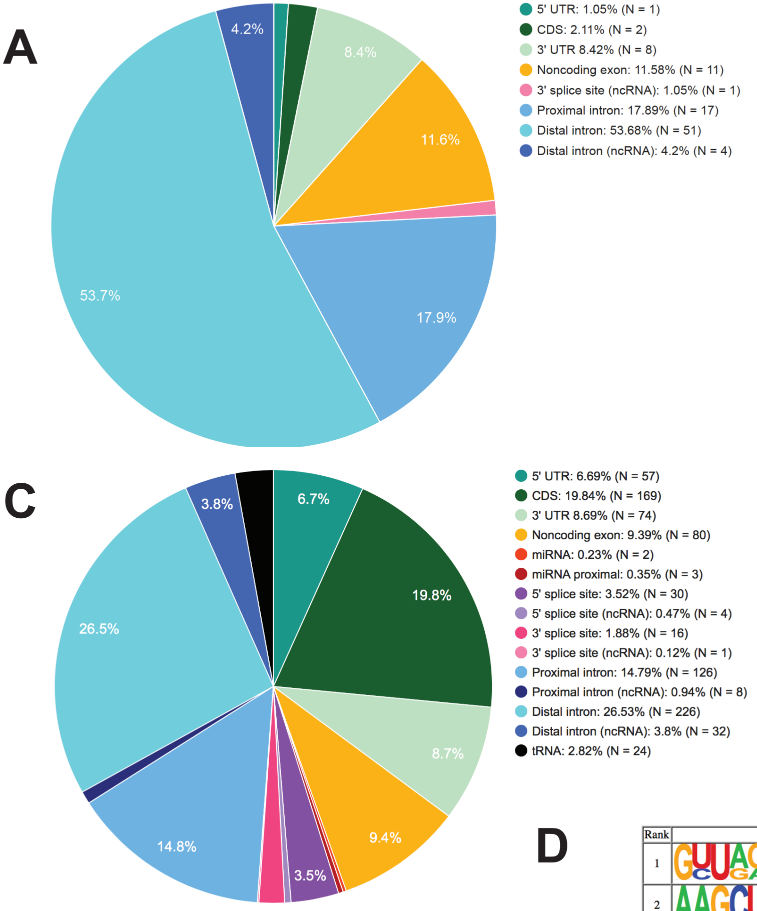

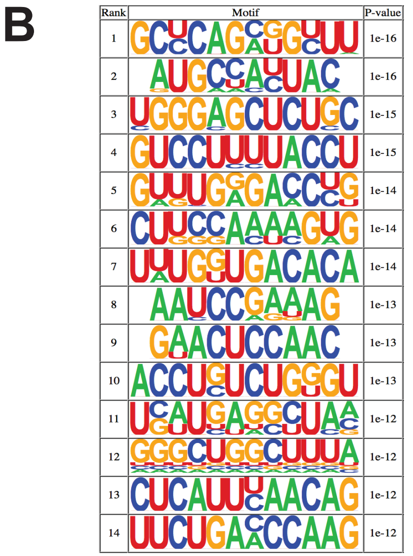

D

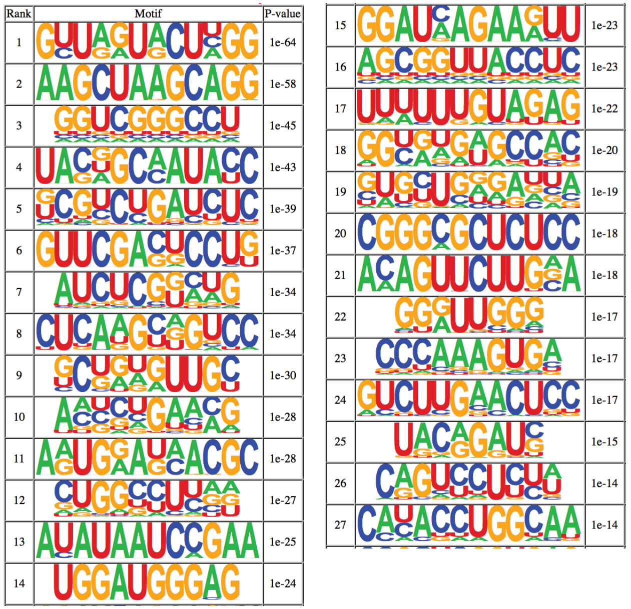

Figure S10. Summary of eCLIP data for Ku deposited in the ENCODE database.
(A) The relative frequency of peaks that map to different RNA sites in HepG2 cells is shown.
(B) RNA Motif analysis of eCLIP data in HepG2 cells shows no clear sequence specificity for Ku-RNA interactions (Total target sequences = 95, total background sequences = 45088).
(C) The relative frequency of peaks that map to different RNA sites in K562 cells is shown.
(D) RNA Motif analysis of eCLIP data in K562 cells shows no clear sequence specificity for Ku-RNA interactions (Total target sequences = 851, total background sequences = 45000). However, several G-rich sequences were identified.

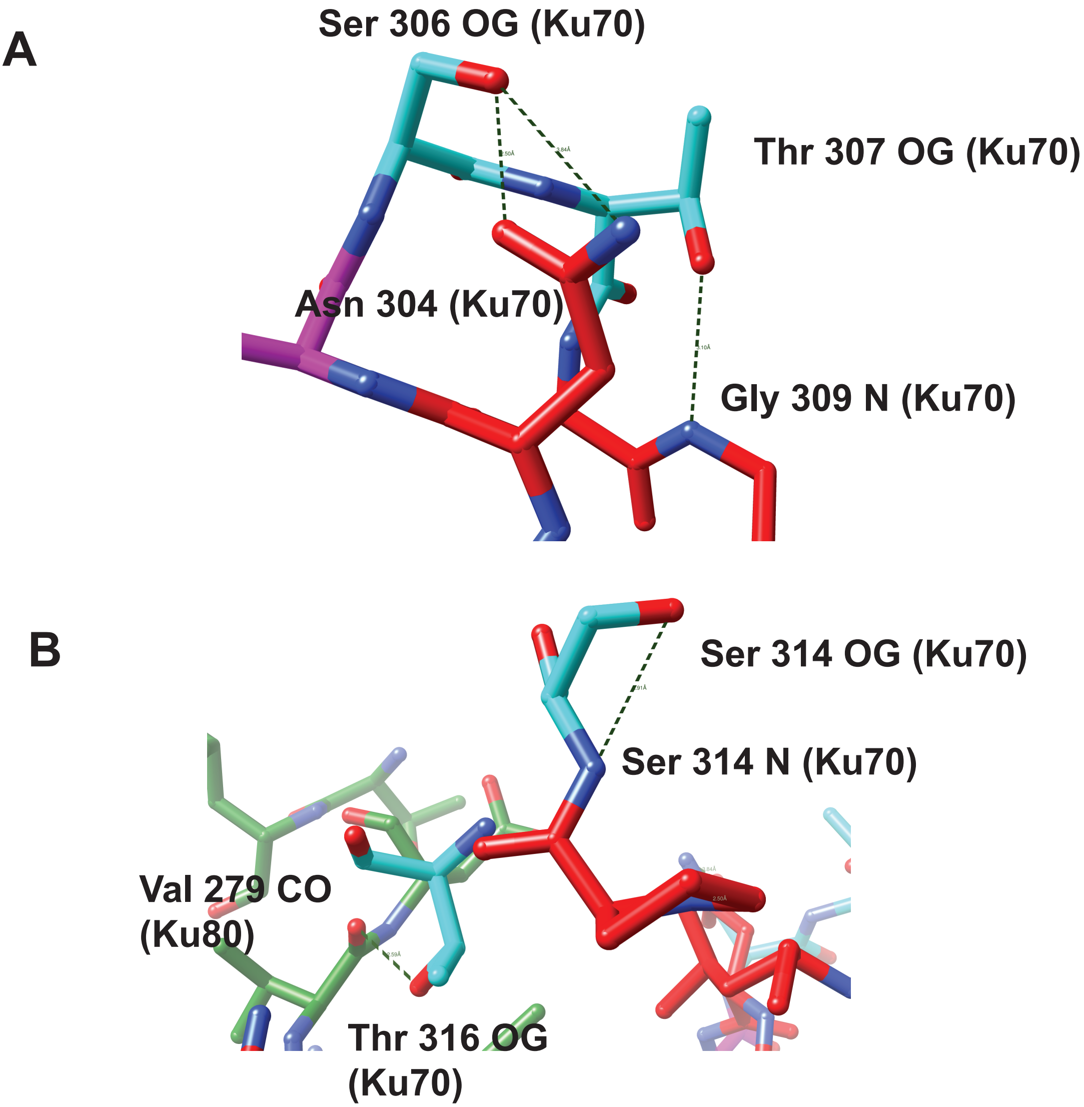

Figure S11. Ser and Thr phosphorylation sites in Ku70 are involved in a network of side chain-side chain and side chain-main chain hydrogen bonding interactions that stabilize the central β-barrel. Loss of this H-bond network due to mutation to alanine or aspartic acid could destabilize the local and global fold of the heterodimer.

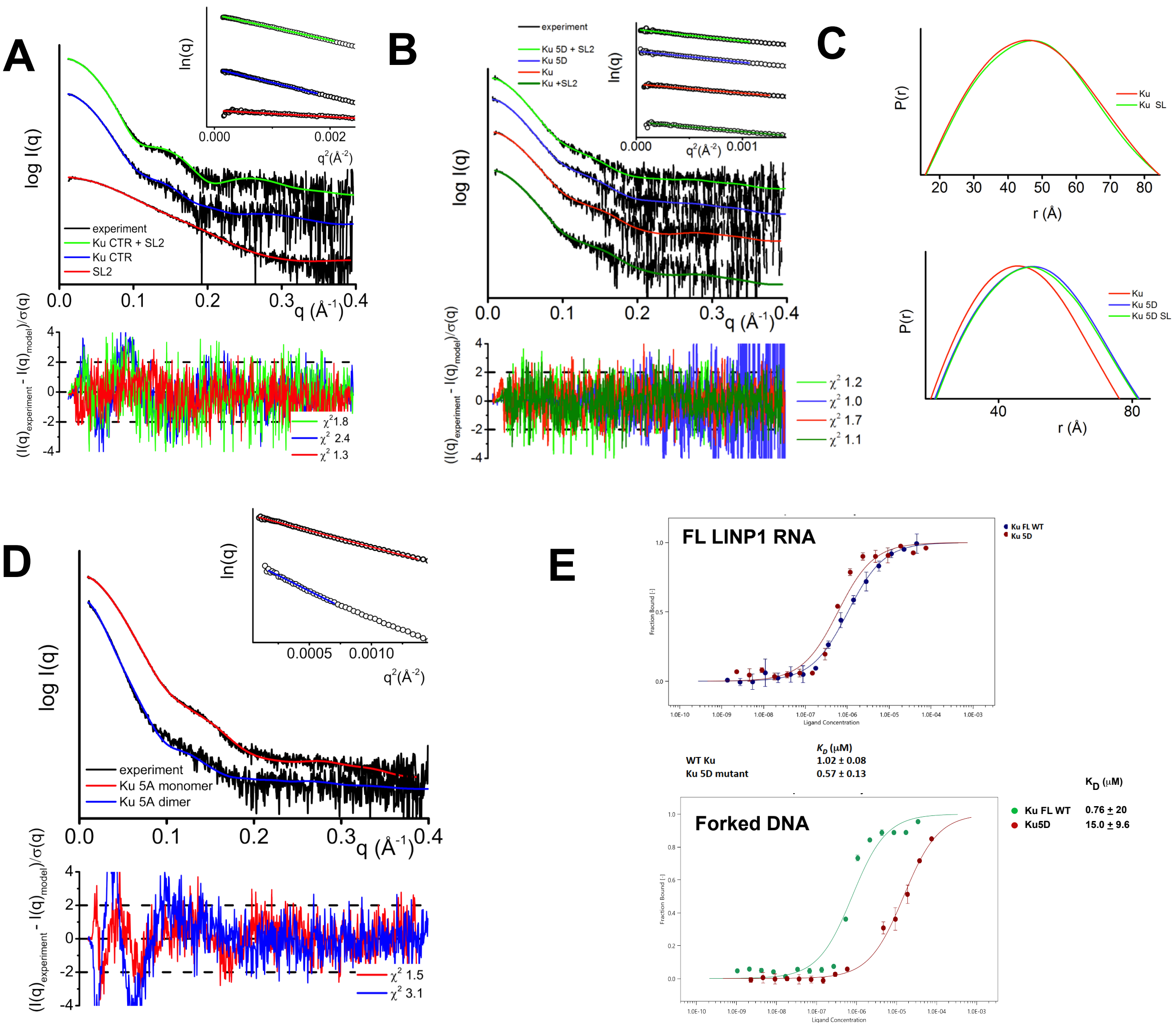

Figure S12. (A) Experimental SAXS profiles (black) of Ku ΔCTR, Ku Δ CTR + SL2 and SL2 and are displayed with the calculated scattering from the atomistic models shown in the main figure 2, together with fit-residuals. Inset shows the Guinier plot of experimental SAXS profiles with the linear fit in the q×Rg < 1.5 limits. (B) Experimental SAXS profiles (black) of Ku, Ku5D, Ku5D + SL2 are displayed with the calculated scattering from the atomistic models shown in the main figure 3, together with fit-residuals. Inset shows the Guinier plot of experimental SAXS profiles with the linear fit in the q×Rg < 1.5 limits. (C) There is a minimal change or narrowing of the P(r) function for both FL Ku and Ku 5D mutant with SL2. (D) Experimental SAXS profiles (black) of Ku5A monomer and Ku5A dimer are displayed with the calculated scattering from the atomistic models shown in the main figure 3, together with fit-residuals. Inset shows the Guinier plot of experimental SAXS profiles with the linear fit in the q×Rg < 1.5 limits. (E) MST binding data for FL Ku WT and FL Ku5D towards LINP1 RNA and Forked DNA substrates.

A

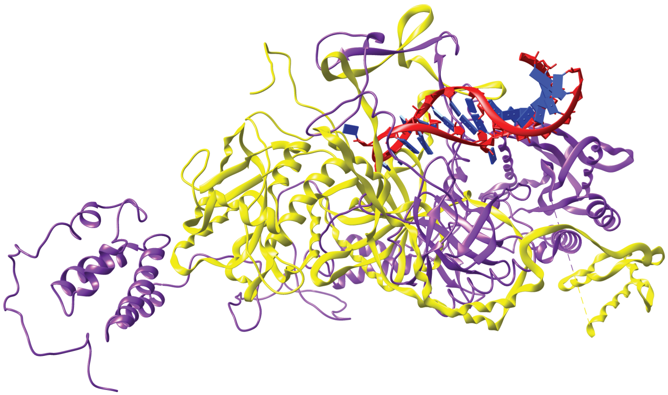

B

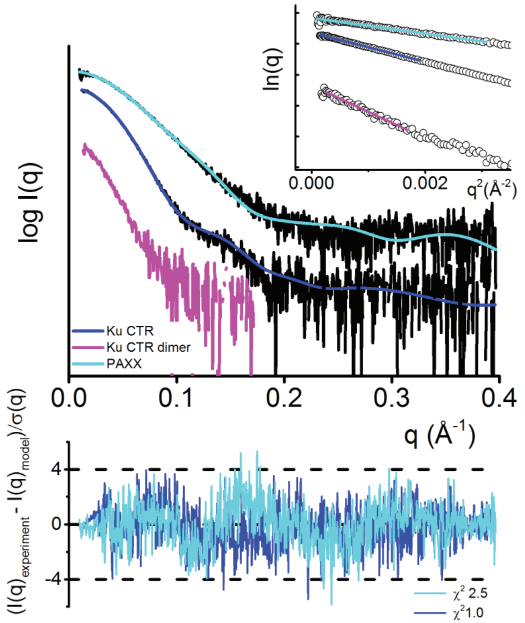

Figure S13. (A) SAXS derived model of FL Ku-SL2 complex. Ku80 is in purple and Ku70 in yellow. (B) Experimental SAXS profiles (black) of PAXX, Ku d CTR monomer and Ku d CTR dimer in the presence of PAXX are shown with the calculated scattering profiles from the atomistic models shown in the main figure 4, together with fit-residuals. Inset shows the Guinier plot of experimental SAXS profiles with the linear fit in the q×Rg < 1.5 limits.

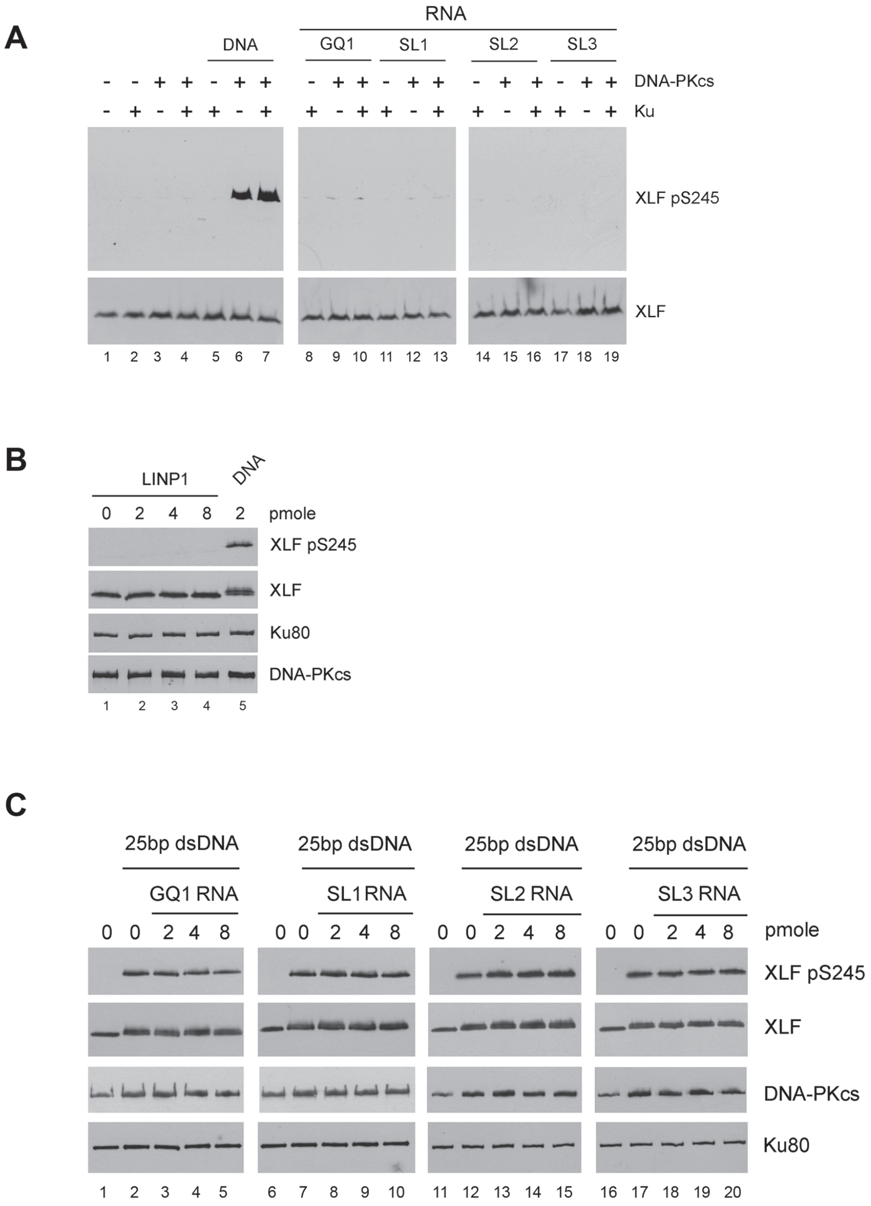

Figure S14. LINP1 RNA does not support DNA-PK kinase activity

(A) LINP1 sub-domains do not support DNA-PK kinase activity. DNA-PKcs (2 pmole) and Ku70/80 (2 pmole) were incubated with XLF under phosphorylation conditions in the presence of 2 pmole 25bp blunt ended dsDNA or 2 pmole GQ1, SL1, SL2 or SL3 RNA as indicated. Reactions were analysed by SDS PAGE and immunblot with antibodies to XLF phospho S245 and XLF as indicated. All blots were developed and exposed at the same time under identical conditions. Note: the Ku independent but DNA dependent phosphorylation of XLF seen in lane 6 was due to overexposure of the gel in order to detect any low levels of RNA dependent phosphorylation. (B) Full-length LINP1 does not support DNA-PK kinase activity. 2 pmole DNA-PKcs and Ku was incubated with 2-8 pmole LINP1 RNA or 25bp DNA as indicated and samples assayed for ability to phosphorylate XLF (S245). (C) LINP1 RNA does not affect DNA-PK dependent phosphorylation of XLF (pS245) in the presence of DNA. 2 pmole DNA-PKcs, and 2 pmole Ku was incubated with 10 pmole XLF and 2 pmole 25 bp DNA under kinase assay conditions. Where incubated, 2, 4, or 8 pmole GQ1, SL1, SL2 or SL3 RNA was added as competitor. Reactions were run on SDS PAGE and immunoblotted for XLF pS245, XLF, DNA-PKcs or Ku80 as indicated. Similar results were seen with full length LINP1 (data not shown).

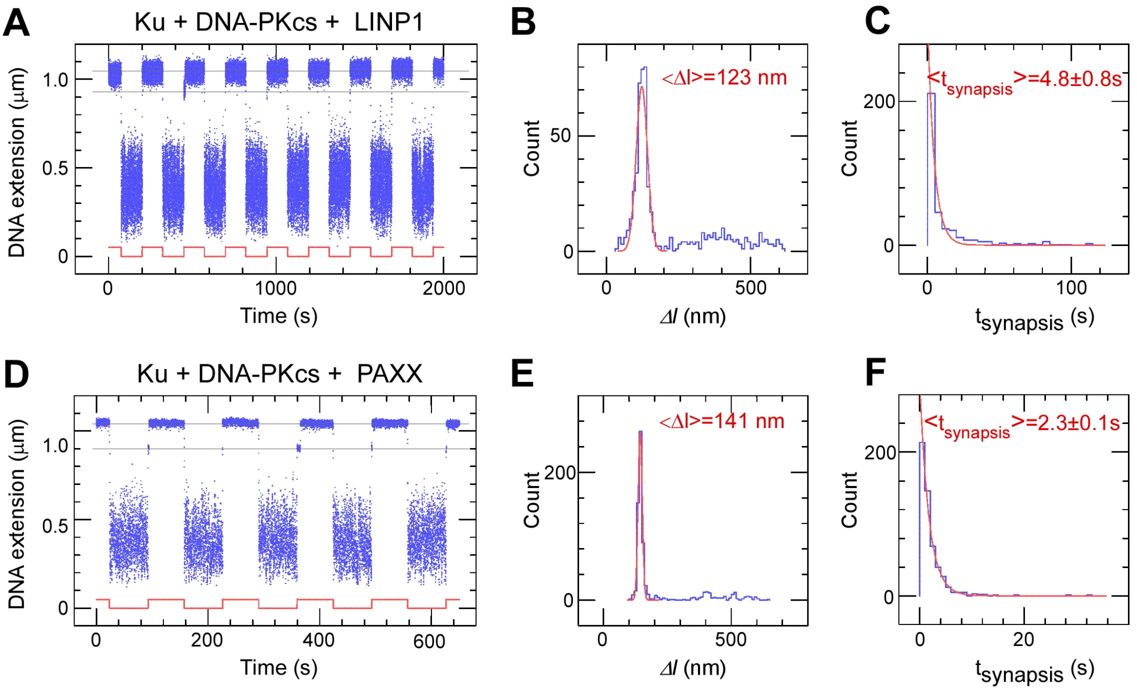

Figure S15. Short Leash LINP1 and PAXX promote DNA-PK-dependent synapsis on a DNA construct with a 600 bp leash. (A) Representative time-trace for Ku + DNA-PKcs + full-length LINP1 obtained upon application of the force-modulation pattern (red). DNA is prepared with blunt ends by SmaI digest. The fourth pulling cycle shows an end-interaction rupture event which can be characterized by both the change in DNA extension upon rupture, Δl, and the duration of the synaptic event prior to rupture, tsynapsis. (B) Histogram of DNA extension change, Δl, upon synapsis rupture in the presence of Ku + DNA-PKcs + full-length LINP1. Red line is fit to a Gaussian distribution, with a peak at 123 nm with 20 nm standard deviation (n=559 events). (C) Lifetime distribution of the synaptic state for Ku + DNA-PKcs + full-length LINP1 is fit to a single-exponential distribution (red line), giving a lifetime of 4.8 ± 0.4 s (SEM, n=357).
(D) Representative time-trace for Ku + DNA-PKcs + PAXX obtained upon application of the force-modulation pattern (red). DNA is prepared with blunt ends by SmaI digest. (E) Histogram of DNA extension change, Δl, upon synapsis rupture in the presence of Ku + DNA-PKcs + PAXX. Red line is a fit to a Gaussian distribution, with a peak at 141 nm and 11 nm standard deviation (n=853 events).
(F) Lifetime distribution of the synaptic state for Ku + DNA-PKcs + PAXX is fit to a single-exponential distribution (red line), giving a lifetime of 2.3 ± 0.1 s (SEM, n=725).

Table S1: Structures Reported For The Ku 70/Ku80 Heterodimer in different DNA/RNA bound complexes.

**Complex**	**Ku70/Ku80**	**Ku70/Ku80 + DNA**	**Ku70/Ku80 + DNA + APLF KBM**	**Ku70/Ku80 + DNA + XLF KBMs**	**Ku70/Ku80 + TLC1 RNA**
PDB code	1jeq	1jey	6erf	6erh (XLF residues 281-299), 6erg (XLF residues 287-299)	5y58
Organism	Human	Human	Human	Human	*S. cerevisiae*
Resolution (Å)	2.7	2.5	3.0	2.8, 2.9	2.8
Affinity (K_d) for DNA/RNA (μM)	-	0.0015 – 0.004	0.0041 ± 0.0007* (-APLF)	0.0041 ± 0.0007* (-XLF)	0.075 ± 0.017
Reference	(Walker et al., 2001)	(Walker et al., 2001)	(Nemoz et al., 2018)	(Nemoz et al., 2018)	(Chen et al., 2018)

Table S2: Sequences of DNAs and RNAs used in this study

Oligo	Sequence
DNA	
25bp duplex	5'-AGC ATT GAC TGG CAT CGT AGC ATC C-3' (25mer)
	5'-GGA TGC TAC GAT GCC AGT CAA TGC T-3'-FAM
d50 duplex	5'-FAM-CTC-TTG-GTG-ATT-ATG-GTT-GCA-ATA-CAT-TTA-ATT-TCA-TTA-TCA-ATT-AG
G-quadruplex	5'-FAM-TTA GGG TTA GGG TTA GGG TTA GGG-3' (24mer)
Fork Substrate	5'-FAM-CGC GCC CAG CTT TCC CAG CTA ATA AAC TAA AAA CTC CTA AGG-3' (42mer)
	5'-CCT TAG GAG TTT TTA GTT TAT TGG GCG CG-3' (29mer)
RNA G-quadruplexes	
LINP1 GQ1	5'-CCU GGG GUA GGA GAG GGU AUG GGG ACC AGG GCA CU-3'
TERRA	5'- UAG GGU UAG GGU-3' (12mer)
RNA Hairpins	
(all with a 5'-FAM)	
SL1	5'-CCC UCC GUC UCC UUG ACU CUG GG-3' (23 mer)
SL2	UGA CUU UUC CUU GCU CAA UAG AAU GAG AAG GAG GUG GCG (39 mer)
SL2-BT	UGA CUG GAG GAA GGA GUA UAG AAA CUC UUC CUU UUG GCG (39 mer)
SL2-LM	UGA CUU UUC CUU GCU CAU UUU UUU GAG AAG GAG GUG GCG (39 mer)
SL3	AAG GCU AGA CCG GGG GCU CGC AUA UCU CCA CUU GCA GCU GCC ACU GCC A (49 mer)
SL3-BT	AAC CGU AGA CCC CGU CGA CCG UUA UCU CCA CUA CGU CGG GGG ACU GGG A (49 mer)
SL3-LM	AA GGC UAG ACC GGG GGC UCG CAU UUC UUU UUU UGC AGC UGC CAC UGC C A (49 mer)
GQ1	CCU GGG GUA GGA GAG GGU AUG GGG ACC AGG GCA CU (35 mer)

SL-H3	UGG CCA AAG GCC CUU UUC AGG GCC ACC C (28 mer)
Oligo RNA tracts	
(all with a 5’-FAM)	
polyA	(A)20
polyC	(C)20
polyU	(U)20
polyG	(G)20

Table S3: Binding affinities from Microscale Thermophoresis (MST) experiments for full length human Ku70/80 and RNAs at 24°C

**RNA/DNA**	**K_D (μm)**	**Fold change relative to dsDNA**
**DNA**		
**d50 DNA**	0.019 ± 0.019	
**RNAs**		
FL LINP1	0.74 ± 0.25	39
LINP1 (1-300)	1.92 ± 0.33	101
SL1	1.69 ± 0.70	89
SL2 - FL Ku	0.9 ± 0.17	47
SL2 - Ku Δ CTR*	1.02 ± 0.18	54
SL2-BT	6.05 ± 9.4	318
SL2-LM	1.62 ± 0.47	85
SL3	0.84 ± 0.45	44
SL3-BT	1.87 ± 0.36	98
SL3-LM	2.37 ± 1.04	125
LINP1 GQ1	0.11 ± 0.04	6
TERRA GQ	0.43 ± 0.18	23
LINP1 SL2 + SL3	0.70 ± 0.17	37
Single stranded RNA oligos (*a*ll with a 5'-FAM)		
polyA	4.8 ± 0.56	252
polyC	1.29 ± 0.44	68
polyU	1.40 ± 0.50	74
polyG	0.17 ± 0.02	9

* The **Ku Δ CTR** was used only for this experiment.

Table S4: Molecular weights, hydrodynamic radii, and particle dimensions determined from SEC-MALS and SAXS experiments

**RNA/ Protein**	Theoretical MW (kDa)	Molecular Mass (MALS) (kDa)	Polydispersity	R_H (MALS) / R_g (SAXS) (Å)	D_{max} (Å)	P_x
FL LINP1	285			N.D.[#]	N.D[#]	2.5 - 2.8
FL-LINP1 (1-300)				N.D.[#]	N.D.[#]	2.3
SL1 RNA	7.7	6.78 (± 2.85%)	1.001 (± 4.08%)	11.0/14.1	45	2.9
SL2 RNA	13.1	11.40 (± 2.09%)	1.007 (± 3.04%)	19.3/17.7	52	3.3
SL3 RNA	16.2	20.26 (± 0.77%)	1.030 (± 1.03%)	40.9/32.3	108	3.4
LINP1 G-quadruplex	12.01	13.60 (± 1.95%)* *monomer peak only	1.089 (± 1.95%)*	20.04*/85.4	245	1.7
Ku70/Ku80 1-569 (ΔCTR)	137.6	161.1 (± 0.54%)	1.001 (± 5.449%)	37.74 (R_g)	120	3.6
Ku70/Ku80 1-569 (ΔCTR) + SL2	150.6			35.85 (R_g)	113	3.8
Ku70/Ku80 1-718 (FL)	154.7	183.7 (± 0.99%)	1.001 (± 5.449%)	41.60 (R_g)	150	3.5
Ku70/Ku80 1-718 (FL) + SL2	167.7	168.1 (± 0.42%)	1.012 (± 1.038%)	42.09 (R_g)	150	3.7
Ku70/Ku80 (1-569) (ΔCTR) dimer	275.2	355.7 (± 3.89%)	1.003 (± 0.760%)	49.83 (R_g)	N.D.	N.D.
Ku70/Ku80 (1-569) (ΔCTR) dimer + SL2	288.2			48.17 (R_g)	N.D.	N.D.
Ku70/Ku80 1-718 (FL) 5D monomer	154.8	196.1 (± 0.294%)	1.000 (± 0.415%)	46.9 (R_g)	137	3.0
Ku70/Ku80 1-718 (FL) 5D monomer + SL2	167.9	209.4 (± 0.060%)	1.005 (± 0.764%)	47.1 (R_g)	122	3.2
Ku70/Ku80 1-718 (FL) 5D dimer	309.65			79.1(R_g)	280	3.2
Ku70/Ku80 1-718 (FL) 5D dimer + SL2	322.65	209.4 (± 0.537%)	1.005 (± 0.764%)	79.0 (R_g)	280	2.8
Ku70/Ku80 1-718 (FL) 5A monomer	154.61			44.87 (R_g)	171.5	3.0
Ku70/Ku80 1-718 (FL) 5A monomer + SL2	167.61			53.57 (R_g)	221.0	3.0
Ku70/Ku80 1-718 (FL) 5A dimer	309.22			62.14 (R_g)	229.0	2.5

Ku70/Ku80 1-718 (FL) 5A dimer + SL2	322.21			58.66 (R_g)	215.0	3.0
FL PAXX	23.82	50.60 (± 1.16%)	1.016 (± 1.62%)	31.05 (R_g)	96.0	3.8
BSA (MALS standard)	66.5, 133, 199.5	62.08, 130.5, 241.8	1.000 for all	-	-	

Not determined due to aggregation in the Gunier region.
